# Ethnic inequalities in multiple long-term health conditions in the United Kingdom: a systematic review and narrative synthesis

**DOI:** 10.1186/s12889-022-14940-w

**Published:** 2023-01-27

**Authors:** Brenda Hayanga, Mai Stafford, Laia Bécares

**Affiliations:** 1 Department of Global Health and Social Medicine, King’s College London, Bush House, North East Wing, 40 Aldwych, London, WC2B 4BG UK; 2 The Health Foundation, 8 Salisbury Square, London, EC4Y 8AP UK

**Keywords:** Multiple long‐term conditions, Ethnicity, Inequalities, Systematic review, Narrative synthesis, UK

## Abstract

Indicative evidence suggests that minoritised ethnic groups have higher risk of developing multiple long-term conditions (MLTCs), and do so earlier than the majority white population. While there is evidence on ethnic inequalities in single health conditions and comorbidities, no review has attempted to look across these from a MLTCs perspective. As such, we currently have an incomplete understanding of the extent of ethnic inequalities in the prevalence of MLTCs. Further, concerns have been raised about variations in the way ethnicity is operationalised and how this impedes our understanding of health inequalities. In this systematic review we aimed to 1) describe the literature that provides evidence of ethnicity and prevalence of MLTCs amongst people living in the UK, 2) summarise the prevalence estimates of MLTCs across ethnic groups and 3) to assess the ways in which ethnicity is conceptualised and operationalised. We focus on the state of the evidence prior to, and during the very early stages of the pandemic. We registered the protocol on PROSPERO (CRD42020218061). Between October and December 2020, we searched ASSIA, Cochrane Library, EMBASE, MEDLINE, PsycINFO, PubMed, ScienceDirect, Scopus, Web of Science, OpenGrey, and reference lists of key studies/reviews. The main outcome was prevalence estimates for MLTCs for at least one minoritised ethnic group, compared to the majority white population. We included studies conducted in the UK reporting on ethnicity and prevalence of MLTCs. To summarise the prevalence estimates of MLTCs across ethnic groups we included only studies of MLTCs that provided estimates adjusted at least for age. Two reviewers screened and extracted data from a random sample of studies (10%). Data were synthesised using narrative synthesis. Of the 7949 studies identified, 84 met criteria for inclusion. Of these, seven contributed to the evidence of ethnic inequalities in MLTCs. Five of the seven studies point to higher prevalence of MLTCs in at least one minoritised ethnic group compared to their white counterparts. Because the number/types of health conditions varied between studies and some ethnic populations were aggregated or omitted, the findings may not accurately reflect the true level of ethnic inequality. Future research should consider key explanatory factors, including those at the macrolevel (e.g. racism, discrimination), as they may play a role in the development and severity of MLTCs in different ethnic groups. Research is also needed to ascertain the extent to which the COVID19 pandemic has exacerbated these inequalities.

**Supplementary Information:**

The online version contains supplementary material available at 10.1186/s12889-022-14940-w.

## Background

Long‐term conditions (e.g. chronic kidney disease, hypertension and depression) are health conditions that are currently not curable and can only be managed with medication or other therapies [1, 2]. One in four primary care patients in the United Kingdom (UK) have multiple long-term health conditions (MLTCs) i.e. the presence of two or more long-term health conditions in an individual [3, 4]. Further, the proportion of people living with four or more long-term conditions is expected to double between 2015 and 2035 [5]. Evidently, healthcare systems, which have previously focused on single conditions, will need to radically transform their approaches to meet the challenges and complexity of caring for people with MLTCs [6].

Indicative evidence suggests that the risk of developing MLTCs is higher, and with MLTCs occurring at an earlier age, for people from minoritised ethnic groups than people from the majority white group [7, 8]. Studies of single conditions provide evidence that many, but not all, people from minoritised ethnic groups in the UK experience poorer health than people from the white ethnic group [9, 10]. Much of the variation in poor health across minoritised ethnic groups is due to underlying socioeconomic inequalities which, in turn, can be attributed to life-course experiences of racism and racial discrimination [11].

With ethnic inequalities evident in single health conditions, we would expect ethnic inequalities in MLTCs to follow a similar pattern. To our knowledge, there has been one previous review that has reported on ethnicity and MLTCs in the UK [12]. However, this review narrowly focused on long-term mental health conditions (i.e. comorbid substance use in psychosis), and therefore, provides only partial evidence of the burden of MLTCs across ethnic groups. Given that having MLTCs is associated with poorer functioning, lower quality of life, higher mortality risk [4], and greater healthcare use and cost [13], developing an understanding of the variation in the burden of MLTCs across ethnic groups in the population is key to ensure efficient, and equitable policy and practice [7].

It is important to consider that there is significant variability in the way ethnicity data is operationalised, analysed and presented in the UK [14]. These variations can introduce bias resulting in incorrect conclusions being drawn, ultimately compromising our understanding of health inequalities [14]. For a complete understanding of ethnic inequalities in the prevalence of MLTCs it is crucial for researchers to carefully consider how ethnicity is best conceptualised and to assess the strengths and limitations of the approaches they adopt during its operationalisation.

Thus, the aim of the present review is 1) to identify and describe the literature that provides evidence of ethnicity and prevalence of MLTCs amongst people living in the UK, 2) to summarise the prevalence estimates of MLTCs across ethnic groups and 3) to assess how ethnicity is conceptualised and operationalised. COVID-19 and the measures adopted to curb the spread of the virus during the pandemic have disproportionately affected minoritised ethnic group people. Given emerging evidence documenting the increase in ethnic inequalities following the coronavirus pandemic, we focus on the state of the evidence prior to, and during the very early stages of the pandemic. This provides a useful benchmark for future studies to measure and understand the impact on the pandemic in further exacerbating longstanding ethnic inequalities in health.

## Methods

### Search strategy

As per the Preferred Reporting Items for Systematic review and Meta‐Analysis Protocols (PRISMA‐P) guidelines [15], we registered the protocol for this review on PROSPERO (CRD42020218061). We electronically searched for studies that included the prevalence of MLTCs across ethnic groups in the UK using the following databases: ASSIA (Applied Social Sciences Index and Abstracts), Cochrane Library, EMBASE (Excerpta Medica dataBASE), MEDLINE, PsycINFO, PubMed, ScienceDirect, Scopus, Web of Science core collection. To ensure that relevant grey literature was not excluded we also conducted a search on OpenGrey. We supplemented the electronic search with a manual search of the reference lists of key articles identified. Experts in the field who had recently conducted systematic reviews on multimorbidity were contacted to compare search strategies and obtain further references. When full texts were unavailable, we contacted the relevant authors.

We adhered to the conventions of each search engine and used search terms that captured the key concepts; Ethnicity (e.g. "Ethnic Groups"[Mesh] OR “BME” OR “BAME”), multiple health conditions (e.g. “Multiple Chronic Conditions” OR Comorbid* OR Multimorbidity), Health inequality (e.g. "Health Equity"[Mesh] OR “Healthcare disparit*” [MeSH] OR Inequalit*) and the geographical location (e.g. "United Kingdom"[MeSH Terms] OR “UK”) (See Supplementary file 1 for a full list of search terms).

### Study inclusion and exclusion criteria

We did not restrict the start of the search to any particular period in time and included studies published up until December 2020. Only UK studies, reported in English, with estimated prevalence of MLTCs across ethnic groups of people in the UK general population, residing in the community were included. To accommodate the variety of ways in which MLTCs were defined and operationalised in the extant literature, we included studies of multimorbidity (i.e. the presence of two or more long-term health conditions [16]) and comorbidity (i.e. the presence of any distinct additional co-existing ailment in a person with an index condition under investigation [17]). We applied further restrictions to address the second objective (to summarise the prevalence estimates of MLTCs across ethnic groups). Given the role of age in patterning MLTCs [2], and the younger age profile of minoritised ethnic group people [18], we excluded studies that did not adjust for at least age as they would have provided an inaccurate representation of the prevalence of MLTCs across ethnic groups. We also excluded studies that focused on only two conditions (e.g. depression and substance abuse) and included only studies that counted more than two conditions. These studies are more likely to focus on people with overall severity of illness, greater healthcare utilisation, and complex medical needs [19, 20].

We imported the studies retrieved from the electronic search to Endnote X8 where duplicates were removed. BH and LB screened a random sample (10%) of the titles, abstracts, and full texts. The studies were divided into 3 batches. For each batch, BH and LB double screened 10% of the studies first and compared results before BH continued to independently screen the remaining studies. Disagreements were resolved by discussion.

### Data extraction strategy

We extracted relevant data from the included studies using a structured form with the following items: study identifier, design, setting, recruitment/data source, sample size, population description, definitions of MLTCs, type and number of MLTCs, confounders, and the results. We denoted any missing information with the acronym NR (i.e. Not Reported). BH and LB double extracted data from a random sample of studies (10%) and reconciled differences through discussion. Thereafter, BH independently extracted data from the remaining studies.

### Population and outcomes

The outcomes of interest were prevalence estimates for MLTCs for at least one minoritised ethnic group, compared to the majority white population.

### Quality appraisal

We assessed the quality of all the studies that contributed to evidence on ethnic inequalities in the prevalence of MLTCs using quality appraisal prompts proposed by Dixon-Woods and colleagues [21] (Supplementary file 2). The prompts can be used to appraise different study designs and focus on aspects of research practice, allowing for the identification and exclusion of studies which are deemed to be fatally flawed [21]. In addition to the quality appraisal, we assessed the relevance of the studies for understanding ethnic inequalities in the prevalence of MLTCs. This approach considers that some papers may have some methodological limitations but may still be of high relevance. Therefore, by considering both methodological quality and relevance, we maximise the inclusion and contribution of a wide range of studies at the level of concepts [21]. This approach is especially relevant given the dearth of studies assessing ethnic inequalities in MLTCs in the UK. All reviewers were involved in the quality appraisal of the studies. MS and LB each appraised a third of the studies. BH appraised the same studies and was in agreement with the ratings allocated by MS and LB (Supplementary file 3). The remaining studies were then independently appraised by BH.

### Data synthesis and presentation

Given the different ways in which MLTCs were conceptualised and operationalised, the different ethnic groups, and the range of conditions explored in the included studies, we conducted a narrative synthesis. We present the findings of the synthesis in themes, supplemented with tables and figures, in two sections. First, we provide an overview of the studies that documented the prevalence estimates of MLTCs across ethnic groups. Second, we present studies where the authors counted more than two long-term conditions and reported on at least age-adjusted prevalence estimates of MLTCs across ethnic groups. Thus far, we have used the term ‘minoritised’ ethnic group people to refer to people who do not self‐identify as belonging to the majority white ethnic group. This term emphasises how social positions are social constructions dependent on context rather than outcomes and practices that are fixed and inevitable [22]. However, as we present the results, we use the terminology used by authors to describe ethnic categories in their studies in recognition of the different labels ascribed to minoritised ethnic group people in the UK.

## Results

### Overview of included studies

**Fig. 1:**
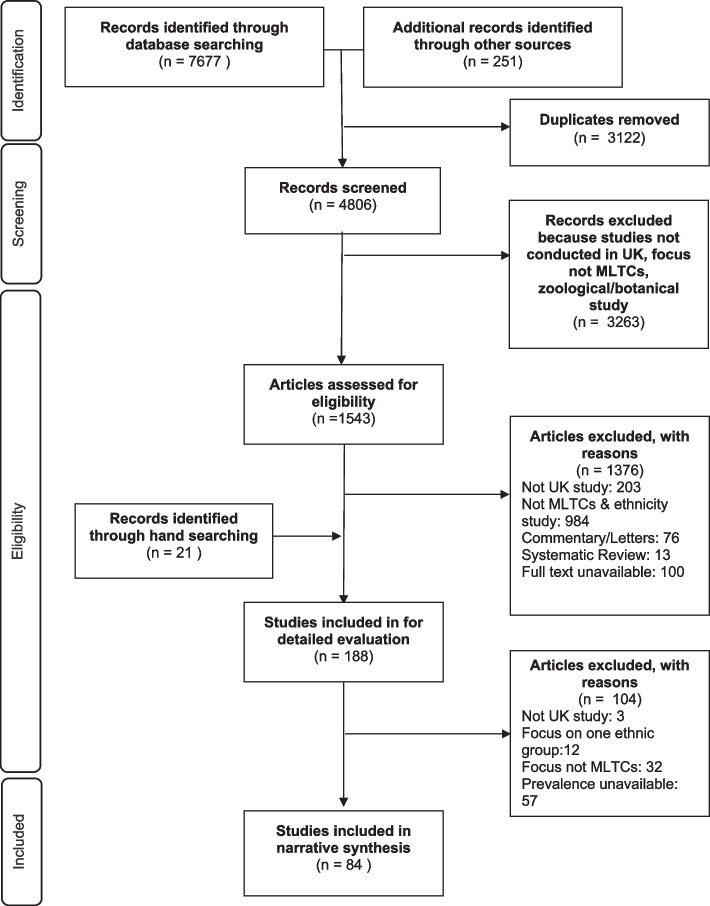
PRISMA Flow chart

We identified 7949 titles from the electronic search, manual search and additional sources (See Fig. 1 which is based on PRISMA guidelines [23]). After removal of duplicates and studies identified as ineligible from the title or abstract, 188 papers were eligible for further evaluation. A further 104 studies were excluded, producing a final sample of 84 studies for the review. Seven of these studies contributed to the evidence of ethnic inequalities in the prevalence of MLTCs among people living in the UK. These were studies in which the authors counted more than two long-term conditions and adjusted for at least age in their analyses.

**Fig. 2:**
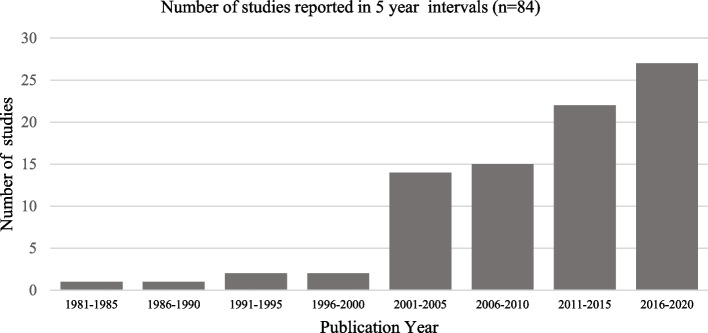
Publication dates of included studies

The 84 studies included were published between July 1984 and October 2020. Figure 2 below illustrates that the number of studies reporting on ethnic differences in MLTCs has increased steadily over the last four decades with a sharp increase in the number of studies published from the year 2001 onwards. The authors of the papers published before the turn of the century conceptualised MLTCs as complications [24], underlying diseases [25] or risk factors [26] for particular conditions. The term comorbidity is used from 2001 onwards to refer to MLTCs and it is not until 2010 that we start to see the use of the term multimorbidity [27].

**Fig. 3:**
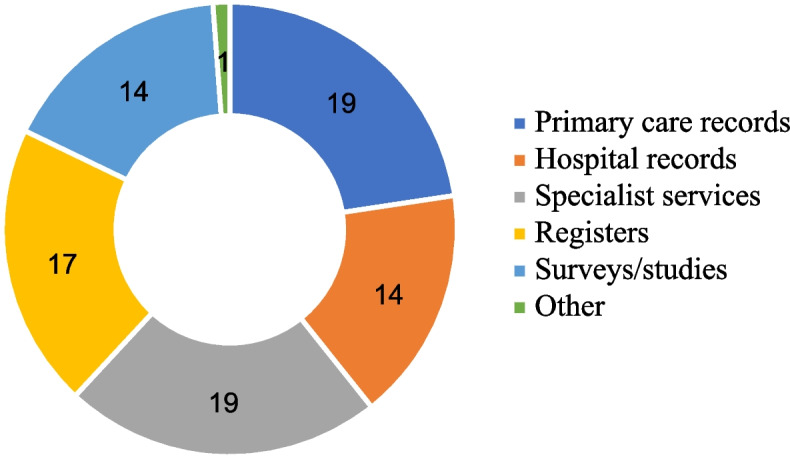
Data Sources used in included studies

In this review, 49 studies were conducted locally, 7 studies were regional, and 28 were national. Sample sizes ranged from 45 to nearly 900,000. The majority of the studies used patient records to analyse the prevalence of MLTCs in people from minoritised ethnic groups (*n* = 69) (Fig. 3). These studies used data from primary care (*n* = 19), hospital records (*n* = 14), specialist clinics/services (*n* = 19) and disease registers (*n* = 17). Fourteen used cross-sectional survey data (e.g. the General Practice Patient survey, Mental Health and Substance Misuse services survey, and the National tuberculosis surveys) and cohort study data (e.g. the HUSERMET Study, the Yorkshire Health Study, the Southall and Brent Revisited study, the Comorbidity Dual Diagnosis Study (*n* = 2), the Millennium Cohort Study (*n* = 2), and UK Biobank (*n* = 4)) [28–41]. In one study, the authors used Scotland-wide linked education and patient databases [42].

### Population characteristics

#### Ethnic group identification

Thirty-eight studies (45%) explicitly reported how ethnicity was identified. Of these, participants self-reported their ethnic identity in 23 studies (27%). One of these studies determined ethnicity based on genealogy [33]. In four studies ethnicity was assigned by interviewers [43], keyworkers [41], researchers, physicians, and nurses [25, 44, 45]. In six studies, ethnicity was both self-reported by participants and clinician assigned [46–51]. Four studies used computerised name recognition programs to identify South Asian people [52–55]. In one study, ethnicity was identified using self-reports, specialist unit ascription, and name recognition software [55].

When ethnicity data could not be identified, some authors attempted to locate the missing information in a variety of ways. One study obtained missing ethnicity data from hospital services and primary care records [56]. Another study imputed the missing participants’ ethnicity from the Census super output area using postcode of residence; where this was an area with ≥ 98% white ethnicity, they assumed the participant was of white ethnicity [55]. The authors in one study relied on the modal ethnicity of patients with the same surname in the Electronic Health Records database where possible [57].

#### Ethnic group categorisation

In this review, 11 studies grouped participants into six or more ethnic group categories and 12 studies used five broad ethnic group categories. The majority of studies (61 out of 84) grouped participants into four ethnic categories or fewer (Supplementary file 5). Of these studies, 19 studies compared the prevalence of MLTCs between two ethnic groups. Table 1 provides a breakdown of the ethnic groups of interest in these studies. Two studies compared Indian [26] and Punjabi people [58] with white European and English people respectively. The remaining 16 studies used broad ethnic minoritised group categories. The majority (*n* = 12) compared the prevalence of MLTCs between Asians/South Asians with white/British/Caucasian/European participants [24, 26, 40, 52–54, 58–63]. Three studies focused on people of Black/Afro-Caribbean and white/Caucasian ethnicity [64, 65]. Four studies categorised participants as white/Caucasian or non-white/other [30, 36, 39, 66].

**Table 1:** Ethnic groups in studies comparing two ethnic group categories

Asian/South Asian ethnicity compared with majority white ethnic group	
Asian	British [24]
Asian	white [59, 60]
Asian	Caucasian [61]
Indian	European [26]
Punjabi	English [58]
South Asian	Caucasian [62]
South Asian	European [40]
South Asian	Europid [63]
South Asian	white European [53, 54]
South Asian	Non-South Asians [52]
**Black/African/Caribbean ethnicity compared with majority white group**	
Afro-Caribbean	Caucasian [65]
Black	white [64, 67]
**Unspecified ethnicity compared with majority white ethnic group**	
Non-Caucasian	Caucasian [39]
Non-white	white [30, 36]
Other	white [66]

In some studies, ethnic categories with small numbers were excluded from the analyses [28, 55, 64, 67–69].

We assessed whether there were changes in how ethnicity was reported over time and we found no discernible pattern. Indeed, studies published in the 80s and 90s used continent/country of origin to ascertain ethnicity [26, 70] or broad ethnic groups such as Asian, Black, Caucasian or British [25, 59, 61]. However, these labels were also observed in more recent studies. Noteworthy is that all the studies that did not specify ethnic group categories and used labels such as non-white, non-Caucasian or other were published between 2016 and 2020 [30, 36, 39, 66]. One study published in 2004 compared white, English-speaking participants self-identifying as English with Asian participants self identifying as Punjabi [58]. They excluded Irish, Scottish or Welsh participants from their analyses as they consider them to be nationally and culturally distinct with different risk profiles from English participants [58].

#### Missing ethnicity data

The percentage of missing ethnicity data was available in 34 studies and ranged from 0.02% [68] to 65% [69]. Missing ethnicity data was treated in various ways. In some studies, those with missing ethnicity data were excluded from the analyses [27, 33, 70] or reported as ‘unknown’ [44]. In other studies, those of unknown ethnicity were combined with those of other ethnicity [31, 37]. Whilst some studies provided the characteristics of those with unknown ethnicity data alongside other ethnic groups [71–73], only two studies specifically conducted sensitivity analyses to ascertain if there were sociodemographic differences between those with missing and complete ethnicity data and discussed the findings [74, 75].

#### Age

The participants’ age was reported in 82 studies. There were 75 studies that included participants aged 16 and above. Of these studies, 31 reported a mean age of 40 years and above. Three studies reported a mean age of between 30 and 39 years [35, 43, 76]. Four studies investigated the prevalence of MLTCs in children [38, 39, 42, 77], and two studies included both adults and children [69, 78].

### Studies reporting on ethnicity and prevalence of MLTCs (Unspecified Index condition)

Of the 84 studies, nine studies counted the number of long-term conditions where there was no specific index condition. The number of ethnic group categories varied across the studies (Table 2).

**Table 2:** Studies reporting MLTCs across ethnic groups with no index condition

**Study ID**	**Study design**	**Geographical reach**	**Data Source**	**Sample size**	**Age in years**	**%Female**	**Ethnic categories**	**Covariates adjusted for in analysis of the prevalence of multiple conditions**
Chudasama et al., 2020 [30]	Longitudinal cohort study	National	UK Biobank	480,940	Age range: 38–73	54	white, Non-white	none
Dorrington et al., 2020 [79]	Longitudinal study	Local	Lambeth Datanet	326,415	Age range: 16–60	53	white, Black African, Asian, Black Caribbean, mixed, other, Black other	age, gender, deprivation
Fleming et al., 2020 [42]	Retrospective study	National	Scotland-wide databases	766,244	Age range: 4–19	49	white, Asian, Black, mixed, other	none
Ashworth et al., 2019 [80]	Longitudinal study	Local	Primary care records	332,353	Age: ≥ 18	50	white, Black, South Asian, mixed, other	age, gender, deprivation, cardiovascular risk
Zemedikun et al., 2018 [29]	Cross-sectional study	National	UK Biobank	502,643	Median Age (IQR): 58 (50–63)	54	white, mixed, Asian, Black, other	none
Hesketh et al., 2016 [38]	Longitudinal study	National	Millennium Cohort Study	9548	Age < 18	51	white, mixed, Indian, Pakistani/Bangladeshi, Black, other	Sex, highest level of maternal education, quintiles of household income
Li et al.,2016 [36]	Longitudinal study	Local	Yorkshire Health Study	27,806	Age range:16–85	56	white, non-white	none
Paddison et al., 2015 [32]	Cross-sectional study	National	General Practice Patient Survey	890,427	Age: ≥ 18	56	white, mixed, Asian, Black, other	none
Mathur et al., 2011 [27]	Cross-sectional study	Local	Primary care records	99,648	Age: ≥ 18	NR	white, South Asian, Black, other	age, sex

### Studies reporting on ethnicity and prevalence of MLTCs (Index condition specified)

The majority of studies reporting MLTCs across ethnic groups specified an index condition (*n* = 75) (Table 3). Measures of comorbidity were used in five studies; three of which used the Charlson Comorbidity Index [69, 71, 81], one used the Elixhauser Comorbidity score [44], and another used the Stoke comorbidity score [82]. As shown in Table 3, kidney-related conditions (*n* = 15), diabetes (*n* = 13), and mental health illnesses (*n* = 12) were the most frequently studied index conditions. COVID-19 was the index condition in three studies [44, 57, 70].

**Table 3:** Studies reporting MLTCs across ethnic groups specifying index condition

**CANCERS**																
**Study ID**	**Study design**	**Geographical reach**	**Data Source**	**Sample size**			**Age in years**		**%Female**		**Ethnic categories**		**Index condition**		**Covariates adjusted for in analysis of the prevalence of multiple conditions**	
Gathani et al., 2020 [81]	Retrospective study	National	National Cancer Registration & Analysis Service data	164,143			Mean Age SD): 59 (11.3)		100		white, Indian, Black Caribbean, Pakistani, Black African		Breast cancer		none	
Samy et al., 2015 [71]	Retrospective study	National	The National Cancer Data Repository	24,361			Age: 83% of sample > 60		46		white, Black, South Asian, other		Myeloma		none	
Nimako et al., 2013 [83]	Retrospective study	Local	Specialist services	423			Mean Age: 64		35		Western European, Afro-Caribbean, Indian Sub-Continent, South East Asian, other		Lung cancer		none	
**DIABETES**																
**Study ID**	**Study design**	**Geographical reach**	**Data Source**	**Sample size**			**Age in years**		**%Female**		**Ethnic categories**		**Index condition**		**Covariates adjusted for in analysis of the prevalence of multiple conditions**	
Mathur et al., 2020 [75]	Observational cohort study	National	Clinical Practice Research Datalink	179,886			Age at diagnosis: 62(13)		45		white, South Asian, Black, other/mixed		Diabetes		age at diagnosis, sex, deprivation	
Mathur et al., 2020b [84]	Observational cohort study	National	Clinical Practice Research Datalink	162,238			Age at diagnosis: 62(13)		45		white, South Asian, Black		Diabetes		none	
Mathur et al., 2018 [85]	Observational cohort study (2006–2016) with nested casecontrol study	local	Primary records	6274			Age range: 25–84		56		white, South Asian, Black		Diabetes		none	
Owusu Adjah et al., 2018 [86]	Matched case control	National	The Health Improvement Network	341,626			Age: ≥ 18		43		white European, African-Caribbean, South Asia		Diabetes		age, sex	
Malavige et al., 2013 [63]	Cross-sectional study	Local	primary care records	510			Mean Age(SD): 57(8)		0		South Asian, Europid		Diabetes		none	
Sivaprasad et al., 2012 [56]	Cross-sectional study	Local	Diabetes register	50,285			Mean Age (SD): 62(15)		47		white European, African/Afro-Caribbean, South Asian, mixed, other		Diabetes		age, gender, region, type of diabetes	
Mehta et al., 2011 [53]	Cross-sectional study	Local	Clinical workstation	5664			Mean Age (SD): 60(17)		46		South Asian, white European		Diabetes		none	
Ali et al., 2009 [54]	Cross-sectional study	Local	The clinical workstation	6230			Age: 55% of sample ≥ 60		46		South Asian, white European		Diabetes		age, gender, comorbidities and complications	
Fischbacher et al., 2009 [52]	Retrospective study	Local	The Diabetes Audit and Research in Tayside	9833			Age:95% of sample > 45		47		South Asian, Non-South Asian		Diabetes		age, sex	
Baskar et al., 2006 [87]	Cross-sectional study	Local	Diabetes register	6047			Age: ≥ 18		46		African Caribbean, Caucasian, Indo-Asian		Diabetes		age, gender, Duration of diabetes (year), BMI, Type 2 Diabetes, Ever smoked, Systolic Blood Pressure	
Earle et al., 2001 [88]	Retrospective case note review	Local	Hospital records	45			Mean Age (SD): 66 (8.5)		36		Indo-Asian, African-Caribbean, Caucasian		Diabetes		age, gender, blood pressure, baseline proteinuria, antihypertensive treatment and smoking habit	
Nagi et al., 1994 [61]	Cross-sectional study	Local	Specialist services	89			Age range: 35–70		24		Caucasian, Asian		Diabetes		none	
Hawthorne et al., 1990 [24]	Cross-sectional study	Local	diabetes register	71			Age: NR		59		Asian, British		Diabetes		none	
**CONDITIONS AFFECTING THE HEART AND BLOOD VESSELS**																
**Study ID**	**Study design**	**Geographical reach**	**Data Source**	**Sample size**			**Age in years**		**%Female**		**Ethnic categories**		**Index condition**		**Covariates adjusted for in analysis of the prevalence of multiple conditions**	
Lawson et al., 2020 [18]	Retrospective study	National	Clinical Practice Research Datalink	108,638			Mean Age (SD): 78(12)		50		white, South Asian, Black		Heart Failure		none	
Gill et al., 2017 [89]	Cross-sectional study	Regional	Primary care records	551			Age range: 40–75		50		white British, African Caribbean, South Asian		hypertension status		none	
Gorantla et al., 2015 [90]	Retrospective study	Regional	Hospital records	42,685			Mean age: 74		49		Caucasian, South Asian, Afro-Caribbean, Oriental, mixed, other		Atrial fibrillation		none	
Sosin et al., 2008 [91]	Cross-sectional study	Local	Hospital records	243			Age: 34% of sample ≥ 65		28		white European, African Caribbean, South Asian		Heart failure		age, gender, diabetes status, smoking status, or BNP vitamin B12, folate, healthy control status	
Patel et al., 2007 [62]	Cross-sectional study	Local	Specialist services	212			Mean Age(SD): 61(13)		33		South Asian, Caucasian		Chronic Heart Failure		none	
Conway et al., 2004 [92]	Retrospective study	Local	Hospital records	388			Mean Age (SD): 70(12)		41		Indo-Asian, Afro-Caribbean, Caucasian		Peripheral artery disease		age, gender	
Blackledge et al., 2003 [73]	Historical cohort study	Local	Hospital records	5789			Age: ≥ 40		50		white, South Asian, other		Heart failure		none	
Lear et al., 1994 [26]	Retrospective study	Local	Specialist services	403			Age range: 33–93		35		Indian, European		Myocardial infarction		none	
Lowry et al., 1984 [59]	Retrospective study	Local	Specialist services	102			Mean Age (SD):50(8)		9		Asian, white		Coronary artery disease		none	
**CONDITIONS AFFECTING THE KIDNEY**																
**Study ID**	**Study design**	**Geographical reach**	**Data Source**	**Sample size**			**Age in years**		**%Female**		**Ethnic categories**		**Index condition**		**Covariates adjusted for in analysis of the prevalence of multiple conditions**	
Steenkamp et al., 2016 [48]	Retrospective study	National	UK Renal Registry	7786			Age: ≥ 18		NR		white, South Asian, Black, other		Renal disease		none	
Hull et al., 2014 [72]	Cross-sectional study	Local	Patient survey	12,011			Mean Age: 71		57		white, South Asian, Black, other		Chronic Kidney Disease		none	
Cole et al., 2014 [64]	Retrospective study	Local	Specialist services	1340			Mean Age (SD): 60(16)		39		white, Black		End stage Renal disease		none	
Jesky et al., 2013 [93]	Retrospective cohort study	Local	Primary care records	31,254			Median age (IQR):59 (50,71)		49		white, South Asian, Black		Chronic Kidney Disease		none	
Rao et al., 2013 [46]	Retrospective study	National	UK Renal Registry	6979			Age: ≥ 18		NR		white, South Asian, Black, other		Renal disease		none	
Udayaraj et al., 2013 [94]	Retrospective study	National	UK Renal Registry	53,565			Age: ≥ 18		38		South Asian, Black, white		Renal disease		none	
Shaw et al., 2012 [47]	Retrospective study	National	UK Renal Registry	5783			Age: ≥ 18		NR		white, South Asian, Black, other		Renal disease		none	
Webb et al., 2011 [50]	Retrospective study	National	UK Renal Registry	4877			Age: ≥ 18		NR		white, South Asian, Black, other		Renal disease		none	
Caskey et al., 2010 [49]	Observational study	National	UK Renal Registry	12,943			Age: ≥ 18		NR		white, South Asian, Black, other		Renal disease		none	
Roderick et al., 2009 [55]	Prospective cohort	National	Renal Registry	5901			Age:82% of sample > 45		38		Caucasian, South Asian, Black		Established Renal failure		age	
Jain et al., 2009 [82]	Prospective study	Regional	Hospital records	755			Age range: 16–86		37		white, Black, South Asian, other		Renal disease		none	
Udayaraj et al., 2009 [51]	Retrospective study	National	UK Renal Registry	11,399			Age: ≥ 18		NR		South Asian, Black, Chinese, other, white		Renal disease		none	
Tomson et al., 2007 [45]	Retrospective study	National	UK Renal Registry	10,579			Age: ≥ 18		NR		South Asian, Black, Chinese, other, white		Renal disease		none	
Bakewell et al., 2001 [60]	Observational study	Local	Specialist services	120			Age: ≥ 18		32		Asian, white		End-stage renal disease		none	
Roderick et al., 1996 [25]	Cross-sectional retrospective study	National	Specialist services	5901			Age: ≥ 16		NR		white, Asian, Black		End stage renal disease		none	
**MENTAL HEALTH CONDITIONS**																
**Study ID**	**Study design**	**Geographical reach**	**Data Source**	**Sample size**			**Age in years**		**%Female**		**Ethnic categories**		**Index condition**		**Covariates adjusted for in analysis of the prevalence of multiple conditions**	
Downs et al., 2017 [77]	Historical cohort study	Local	Clinical Record Interactive Search system	638			Age range: 10–17		50		white British, white Other, Black, Asian, Mixed		Psychosis		none	
Nicholl et al., 2015 [28]	Cross-sectional study	National	UK Biobank	144,139			Mean age (SD): 57(8.1)		53		white, Asian, Black		Depression		age, sex, quintiles of Townsend score, smoking status, frequency of alcohol consumption, BMI, morbidity count	
Bruce et al., 2012 [76]	Cross-sectional study	Local	Specialist services	165			Mean age (SD): 39(10)		0		African, Caribbean, white British		Severe mental illness		none	
Mazzoncini et al., 2010 [95]	Retrospective study	Local	Specialist services	511			Age range: 16–64		41		white British, white other, Black Caribbean, Black African, Asian, other		Psychosis		none	
Pinto et al., 2010 [67]	Cross-sectional study	Local	Lambeth DataNet	903			Age range: 16–74		40		white, Black		Psychosis		age, gender, obesity and deprivation	
Afuwape et al., 2006 [35]	Retrospective cohort study	Local	COMO study	213			Mean Age: 37		16		white, Black Caribbean, Black African, Black British		Psychotic illness		age	
Bhui et al., 2004 [58]	Cross-sectional study	Local	Primary care records	389			Age: ≥ 16.5		56		Punjabi, English		Depression		age, gender, social class, employment, marital status, number of body systems affected by physical illness	
Kennedy et al., 2004 [96]	Epidemiologically-based study	Local	Specialist services	246			Age: > 16		54		white European, African–Caribbean, African, other		Severe mental illness		none	
Miles et al., 2003 [34]	Cross-sectional study	Local	COMO study	214			Age range: 17–77		17		white, Black, other		Psychotic illness		none	
McKenzie et al., 2002 [43]	Cross-sectional design	Local	Hospital records	337			Mean Age(SD): 33 (13)		44		white, African-Caribbean, Mixed African-Caribbean and white, African, South Asian, Other		Psychosis		age, sex, social class, RDC diagnosis and referral status	
Graham et al., 2001 [41]	Cross-sectional study	Local	Mental Health and Substance Misuse services survey	498			Age range: 18–71		22		white UK, African-Caribbean, Asian, European, Irish, mixed, Black other, other		Severe mental illness		none	
Weaver et al., 2001 [97]	Cross-sectional study	Local	Specialist services	851			Mean Age(SD): 45(14)		44		white UK/European, Black, Irish, Other		Psychotic disorders		age, sex, diagnosis	
**NEUROLOGICAL CONDITIONS**																
**Study ID**	**Study design**	**Geographical reach**	**Data Source**	**Sample size**			**Age in years**		**%Female**		**Ethnic categories**		**Index condition**		**Covariates adjusted for in analysis of the prevalence of multiple conditions**	
Bhanu et al., 2020 [74]	Retrospective cohort study	National	The Health Improvement Network database	10,570			Mean Age: 80		64		white, Asian, Black		Dementia		none	
Taylor et al., 2020 [31]	Longitudinal cohort study	National	UK Biobank	1568			Age range: 40–69		72		white, BAME, other/unknown		Multiple sclerosis		none	
Jordan et al., 2017 [39]	Longitudinal study	National	Millennium Cohort Study	7224			Age: < 18		49		Caucasian, Non-Caucasian		Dyslexia		none	
Liew et al., 2016 [98]	Retrospective study	Regional	Hospital records	3275			Mean Age: 71		53		Caucasian, South Asian, Afro-Caribbean, Oriental, mixed, other		Transient ischemic attack		none	
Eastwood et al., 2015 [40]	Longitudinal study	Local	The SABRE study	4196			Age range: 45–58		0		South Asian, European		Stroke		age, smoking status, waist/hip ratio, total/HDL-cholesterol ratio, diabetes mellitus, fasting glucose, physical activity, heart rate	
Potluri et al., 2015 [99]	Retrospective study	Regional	Hospital records	17,415			Mean Age: 74		50		Caucasian, South Asian, Afro-Caribbean, Oriental, mixed, other		Ischaemic stroke		none	
Sarker et al., 2008 [100]	Prospective study	Local	The South London Stroke Register	566			Mean Age(SD): 62(17)		46		white, Black, other/missing		Haemorrhagic stroke		none	
Patel et al., 2001 [101]	Prospective study	Local	South London Stroke Register	235			Mean Age(SD): 71(14)		51		white, Black, other		Stroke		none	
**RESPIRATORY CONDITIONS**																
**Study ID**	**Study design**	**Geographical reach**	**Data Source**	**Sample size**			**Age in years**		**%Female**		**Ethnic categories**		**Index condition**		**Covariates adjusted for in analysis of the prevalence of multiple conditions**	
Sapey et al., 2020 [57]	Observational cohort study	Local	Hospital records	2217			Median Age(IQR): 73(58–84)		42		white, mixed, South Asian/South Asian British, Black/African/Caribbean/Black British, other		COVID-19 infection		none	
Zakeri et al., 2020 [70]	Cohort observational study	local	Lambeth DataNet	872			Age: ≥ 18		44		Black, white, mixed/other, Asian		COVID-19 infection		none	
Perez-Guzman et al., 2020 [44]	Retrospective study	Local	Hospital records	559			Median Age(IQR): 69(25)		38		white, Black, Asian, other		SARS-CoV-2 infection		none	
Marshall et al., 1999 [102]	Retrospective study	Local	Specialist services	157			Age:94% of sample > 20		34		European, African, Asian, Americans		Tuberculosis		none	
**OTHER HEALTH CONDITIONS**																
**Study ID**	**Study design**	**Geographical reach**	**Data Source**	**Sample size**			**Age in years**		**%Female**		**Ethnic categories**		**Index condition**		**Covariates adjusted for in analysis of the prevalence of multiple conditions**	
Ali et al., 2020 [103]	Observational study	Regional	Specialist services	313			Mean Age(SD): 52(11)		25		Asian/Asian British, white, Black/African/Caribbean/Black British, mixed, other ethnic group		Hepatitis C infection		none	
Eendebak et al., 2017 [33]	Cross-sectional observational study	Local	HUSERMET Study	642			Age range: 40–48		0%		South Asian, white European, African Caribbean		Reproductive hormone levels		none	
Misra et al., 2016 [69]	Retrospective cohort study	National	Hospital records	71,966			Age range:2–104		52		White European, Indian, Pakistani, Bangladeshi, Black, Chinese,		Ulcerative colitis		none	
Patel et al., 2016 [66]	Cross-sectional observational study	Local	Specialist services	299			Mean Age(SD): 58(2)		5		white, other		HIV		none	
Nisar et al., 2015 [104]	Retrospective study	Local	Specialist services	299			Mean Age at start of therapy:51		64		Caucasian, Asian, Afro-Caribbean, Mixed		Inflammatory arthritis		none	
Chackayil et al., 2013 [105]	Retrospective, cross-sectional study	Regional	Hospital records	3563			Age: NR		59		white, South Asian, African Caribbean		Iron deficiency Anaemia		none	
Kitley et al., 2012 [65]	Retrospective study	Local	Specialist services	106			Mean Age at onset:41		87		Caucasian, Afro-Caribbean, Japanese		Neuromyelitis optical		none	
Mann et al., 2008 [106]	Retrospective	National	Hospital records	40,488			Mean Age(SD): 42(13)		39		white, Black African, Black Caribbean, Pakistani, Bangladeshi, Other, Mixed, Indian, Chinese		Hepatitis C		age, sex, year	
Mohsen et al., 2005 [68]	Cross-sectional design	Local	Specialist services	1017			Median age at presentation(IQR):32.6(29–38)		35		white, Black African, Black Caribbean, Black Other, South Asian, Other, Oriental		HIV		gender, HIV risk group, Place of birth, History of Injecting Drug Use, Blood factor	
Dragovic et al., 2002 [78]	Retrospective study	Local	Specialist services	153			Age: 97% aged ≥ 17		19.6		white, Black Caribbean, Black African,		Gonococcal infection		age, sex	
Rose et al., 2002 [37]	Retrospective study	National	National TB survey database & HIV/AIDS patient database	3432			Age range: 16–54		45		white, Black African, Indian sub-continent, other/unknown		HIV		year, sex, geographical area	

### Prevalence of multiple long-term conditions by ethnic group

Seven studies provided evidence on ethnicity and age-adjusted prevalence of multiple conditions amongst people living in the UK (Table 4). Of these, three were studies of MLTCs with no specified index condition [27, 79, 80]. All seven studies were appraised and were considered to be of high methodological quality (Table 4, Supplementary file 4). In all studies, the authors clearly specified the study designs which we deemed to be appropriate to address the aims and objectives of the research. There was a clear description of the methods of analysis and the process by which the results were produced. In addition, the interpretations and conclusions of the authors were supported by the data they presented. The findings of these studies suggest that people from minoritised ethnic groups have a higher prevalence of MLTCs when compared to people from the majority white group [27, 79, 80]. All were local studies that used patient records in their analyses. All adjusted for age and gender/sex, with two studies also adjusting for deprivation [79, 80] and one also adjusting for cardiovascular risk factors [80]. Mathur and colleagues investigated the cardiovascular multimorbidity by ethnicity in a socially deprived and multi-ethnic population in east London [27]. Their focus was on five health conditions: coronary heart disease, diabetes, heart failure, stroke, and hypertension across four ethnic groups. After adjusting for age and sex, and clustering by general practice, minoritised ethnic group people were more likely to have MLTCs than white people with adjusted odds ratios (OR) of 2.4 (95% Confidence interval (CI): 1.94–2.15) for South Asian people and 1.23 (95% CI: 1.18–1.29) for Black people [27].

**Table 4:** Studies reporting adjusted prevalence of MLTCs multiple conditions across ethnic groups

**Study ID**	**Region**	**Data Source**	**Sample size**	**No. of Ethnic Categories**	**No. of conditions**	**Types of conditions**	**Index condition**	**Covariates**	**Effect measure** ORs/Prevalence and 95% CI Reference: White Ethnicity	**Overall Study Quality**
Dorrington et al., 2020 [79]	Local	Lambeth Datanet	326,415	Asian, Black African, Black Caribbean, Black other, mixed, other, white	13	Epilepsy, Chronic Pain, Depression, Vascular, Cancer, Cardiac Disease, Rheumatoid Arthritis, Severe Mental Illness, Hypertension, Diabetes, Obesity, Respiratory Disease, Learning Disability	-	age, gender, deprivation	Black African: 1.92 (1.75–2.10) Black Caribbean: 2.83 (2.56–3.14) Mixed: 1.50 (1.31–1.72) Black Other: 2.22 (1.87–2.64)	High
Mathur et al., 2020 [84]	National	CPRD data	179,886	Black, South Asian, white	8	Diabetes, Hypertension, Coronary Heart Disease (Angina/Myocardial Infarction), Heart Failure, Chronic Kidney Disease, Retinopathy, Neuropathy	Diabetes	age, sex, deprivation	South Asian: 0.88 (0.80–0.96) Black: 0.50 (0.43–0.58)	High
Ashworth et al., 2019 [80]	Local	Primary care records	332,353	Black, mixed,other, South Asian, white	12	Atrial Fibrillation, Chronic Obstructive Pulmonary Disease, Chronic Pain, Chronic Kidney Disease, Coronary Heart Disease, Diabetes Mellitus, Dementia, Depression, Heart Failure, Serious Mental Illness, Stroke, Morbid Obesity	-	age, gender, deprivation, cardiovascular risk	South Asian: 1.44 (1.29–1.61) Black: 0.86 (0.80–0.92)	High
Owusu Adjah et al., 2018 [86]	National	THIN data	341,626	African-Caribbean, South Asian, Western European	5	Diabetes, Myocardial Infarction, Heart Failure, Stroke, Cancer	Diabetes	age, sex	*Normal BMI* W. Europeans: 10.4% (9.5, 11.3) South Asians: 6.8% (4.8, 9.5) African-Caribbeans: 6.4% (4.0, 10.4)	High
Mathur et al., 2011 [27]	Local	Primary care records	99,648	Black, other, South Asian, white	5	Hypertension, Ischaemic Heart Disease, Heart Failure, Stroke, And Diabetes	-	age, sex	South Asian: 2.4 (1.94–2.15) Black: 1.23 (1.18–1.29)	High
Roderick et al., 2009 [55]	National	UK Renal Registry data	5901	Black, South Asian, Caucasian	8	Established Renal Failure, Coronary Heart Disease, Diabetes, Any Vascular Disease, Chronic Obstructive Pulmonary Disease, Smoking, Any Malignancy, Chronic Liver Disease	Established Renal Failure	age	South Asian: 1.26 (1.04–1.52) Black: 0.70 (0.52–0.95)	High
Baskar et al., 2006 [87]	Local	Diabetes Register	6047	Afro-Caribbean, Caucasian, Indo-Asian	6	Diabetes, Retinopathy, Dipstick Proteinuria, Ischaemic Heart Disease, Cerebrovascular Disease, Peripheral Vascular Disease	Diabetes	age, gender, diabetes complication risk factors	*Microvascular complications:* Afro-Caribbean: 1.29 (1.06–1.57) *Macrovascular complication:* Afro-Caribbean: 0.71(0.51–0.87) Indo-Asian: 0.81 (0.67–0.93)	High

*OR* Odds Ratio, *CI* Confidence Interval, *THIN* The Health Improvement Network, *CPRD* Clinical Practice Research Datalink

Ashworth and colleagues examined the social determinants and cardiovascular risk factors for multimorbidity and the acquisition sequence of multimorbidity [80]. Their focus was on 12 conditions across five ethnic groups. Age-sex adjusted estimates were not provided but they found that compared to white people, Black and South Asian people had higher odds of multimorbidity after adjusting for age, gender, and area level deprivation (OR 1.15 (95% CI: 1.07–1.23) and 1.19 (95% CI: 1.07–1.33) respectively). However, when hypertension, obesity, and smoking status were added to the model, only South Asian people had higher odds of multimorbidity (1.44 [95% CI: 1.29–1.61]), but not Black people (0.86 [95% CI: 0.80–0.92]) [80].

Dorrington and colleagues assessed 13 conditions across seven ethnic groups. Age-sex adjusted estimates were not provided [79]. Age, gender and area-level deprivation adjusted models showed that the odds of having two or more long-term conditions were higher in some minoritised ethnic groups; 1.92 (95% CI: 1.75–2.10) for Black African people, 2.83 (95% CI: 2.56–3.14) for Black Caribbean people, 1.50 (95% CI: 1.31–1.72) for people with mixed ethnicity and 2.22 (95% CI: 1.87–2.64) for people who self identified as Black other. When three or more long-term conditions were considered, the same minoritised ethnic group populations still had higher odds of multimorbidity than the white population (OR 3.42 [95% CI:3.05–3.83] for Black Caribbean people, 2.53 [95%CI: 2.02–3.18] for Black other people, 1.95 [95% CI 1.74–2.20] for Black African people and 1.50 [95% CI: 1.30–1.73] for people with mixed ethnicity) [79]. In the remaining four studies, an index condition was specified [55, 75, 86, 87]. Two studies, one which focused on people starting renal replacement therapy, and the other which focused on people with diabetes, reported a higher prevalence of MLTCs in people from some minoritised ethnic groups [89, 48]. The other two studies which focused on people with diabetes, reported lower prevalence of MLTCs among minoritised people [75, 86]. Three out of these four studies used national data [55, 75, 86]. All used patient records to assess MLTCs across three ethnic group categories: Black/African-Caribbean, South Asian/Indo-Asian and white ethnicity. All four studies adjusted for age, three studies additionally adjusted for sex [75, 86, 87]. One study additionally adjusted for area-level deprivation [75] and another for diabetes complication risk factors [87].

In their study exploring the survival of patients starting renal replacement therapy across three ethnic groups, Roderick and colleagues found that compared to white patients, the age-adjusted prevalence of vascular comorbidity was higher in South Asian patients but lower in Black patients [odds ratios of 1.26 (95% CI:1.04–1.52) and 0.70 (95% CI:0.52–0.95) respectively]. However, other comorbidities were found to be generally more common in white patients [55]. Baskar and colleagues evaluated ethnic differences in the prevalence of hypertension and vascular complications in a population with diabetes [87]. They considered microvascular complications to be the documented presence of any grade of retinopathy and/or dipstick proteinuria [87]. Macrovascular complications were considered to be the documented presence of ischaemic heart disease and/or cerebrovascular disease, and/or peripheral vascular disease [87]. Age-adjusted estimates were not provided. After adjusting for age, gender, BMI, systolic blood pressure, smoking, type 2 diabetes, and duration of diabetes, Afro-Caribbean people had a higher risk of microvascular complications (odds ratios of 1.293 (95% CI: 1.063–1.573) relative to white people). However, compared to white people, both Afro-Caribbean and Indo-Asian people had significantly lower risk of macrovascular complications with odds ratios of 0.710 (95% CI: 0.581–0.866) and 0.807 (95% CI: 0.669–0.933) respectively [87].

Owusu-Adjah and colleagues examined ethnic differences in comorbidities in patients with type 2 diabetes mellitus [86]. They found that Western European patients had significantly higher baseline age-sex adjusted prevalence of cardiovascular complications compared to South Asian patients (at all levels of BMI) and African-Caribbean patients (in overweight or obese groups only). Western Europeans also had significantly higher baseline prevalence of cancer and depression [86]. Mathur and colleagues investigated ethnic differences in the severity and clinical management of type 2 diabetes at initial diagnosis. Age-adjusted estimates were not provided. Adjusting for age, sex and deprivation, and clustering by practice, the odds of having comorbid macrovascular disease (i.e. hypertension, coronary heart disease (including myocardial infarction and angina), stroke, and heart failure) at diagnosis were reduced in South Asian people (0.88, 95%CI 0.80–0.96) and halved in Black people (0.50, 95%CI 0.43–0.58) relative to white people. However, they found no ethnic differences in the odds of having diagnosed microvascular disease (i.e. chronic kidney disease, retinopathy, and neuropathy) in their sample [75].

## Discussion

We identified seven studies that give insight into age-adjusted ethnic differences in the prevalence of MLTCs. The findings are indicative of ethnic inequalities in MLTCs in favour of the majority white as five of the seven studies reported that some minoritised ethnic groups have a higher prevalence of MLTCs than their white counterparts. The evidence suggests that South Asian people (three out of five studies) and Black people (two out of five studies) may be at a higher risk of MLTCs [27, 55, 79, 80, 87]. Whilst some studies adjusted for factors that may be on the explanatory pathway, including deprivation and risk factors for cardiovascular disease and diabetes complications, all studies adjusted for at least age in their analyses. As such, the evidence of ethnic inequalities in MLTCs is based on studies that considered this key confounder [2]. However, given the variation in the number and types of conditions examined in these studies and the merging of different minoritised ethnic groups, this evidence may not accurately reflect the true level of inequality.

The two studies that reported a lower prevalence of MLTCs in minoritised ethnic groups are recent studies which focused on people living with diabetes. This finding is intriguing and warrants further attention. In trying to understand these observations, we cannot rule out errors with measurement of ethnicity and data quality. We must also consider that Black and South Asian people in the UK not only have a higher prevalence of diabetes, but they also develop the condition at an earlier age [84, 107]. It is, therefore, possible that the minoritised populations included in these studies are younger than their white counterparts. Since MLTCs increase with age, the lower prevalence of MLTCs among minoritised ethnic groups observed in these studies could result from residual confounding by age. Otherwise put, there may be persistent differences in age among minoritised ethnic group people and white people even after controlling for age. Also, given that minoritised ethnic groups have a higher risk of developing diabetes, much effort might be paid to the identification and management of diabetes in this population. Mathur and colleagues, who examined the ethnic variations in the severity and management of diabetes at first diagnosis, provide support for this notion [75]. They found that when compared to white people, Black and South Asian people had better capture of risk factors, better/similar cardio-metabolic profile at diagnosis, faster initiation of anti-diabetic treatment, first National Health Service (NHS) health check and structured education [75]. These outcomes may prevent further health problems, thereby, contributing to a lower prevalence of MLTCs in minoritised people with diabetes. However, other studies report ethnic inequalities in diabetes care with Black and Asian people found to have worse glycaemic control and being less likely to be prescribed newer therapies [108]. Inequalities in diabetes care can result in higher rates of complications [108] which can increase the likelihood of MLTCs. Future work is, therefore, required to explore these findings further.

### Proposed mechanisms underlying observed inequalities

Given that few studies have assessed the prevalence of MLTCs across ethnic groups, it is difficult to ascertain the reasons behind the observed ethnic inequalities in the prevalence of MLTCs in the UK. However, based on longstanding international evidence on ethnic inequalities in single conditions, we propose a number of mechanisms. We consider the impact of racism and discrimination in explaining the observed ethnic differences in the prevalence of MLTCs as they are known to influence health [109]. It is possible that racism and multiple forms of discrimination can intersect with demographic factors (e.g. age, gender, and/or sexual orientation) resulting in disadvantage in accessing key economic, physical and social resources for some, thereby, leading to socioeconomic and health inequalities [110–112]. In turn, these inequalities can result in higher prevalence of some health conditions which may then accelerate the development of MLTCs in some minoritised ethnic group populations. Racism and multiple forms of discrimination can also lead to ethnic inequalities in healthcare access, utilisation and care quality [113] through a number of pathways. For example, findings from international studies suggest that negative discriminatory practices can result in mistrust of healthcare professionals, non-compliance with treatment, delayed diagnoses and treatment and even forgone healthcare [114–116]. These outcomes are detrimental as they not only exacerbate existing health inequalities, but they can also lead to the development and/or progression of MLTCs. However, these processes have received very little investigation in the context of ethnic inequalities in the prevalence of MLTCs in the UK.

### Quality of studies contributing to ethnic inequalities in MLTCs

We assessed the quality of the seven studies which contributed to the evidence on ethnic inequalities in the prevalence of MLTCs and considered the studies to be of high quality. Whilst the studies were methodologically sound, we identified limitations which potentially impede a full understanding of ethnic inequalities in the prevalence of MLTCs. For example, in one study, ethnicity, deprivation, and socially-patterned risk factors were considered in isolation [80]. Relatedly, some authors presented only fully adjusted estimates [79, 87] which doesn’t allow for the assessment of the impact of key variables on the prevalence of MLTCs. We noted the use of broad ethnic categories, patient records and a general lack a sensitivity analysis to assess the characteristics of those with and without complete ethnicity data. We discuss these limitations and their implications for understanding ethnic inequalities in MLTCs in the following section.

#### Broad ethnic group categories

In the seven studies that contributed to the evidence of ethnic inequalities in the prevalence of MLTCs, people from minoritised ethnic groups were often grouped into broad categories; in particular, Black/African-Caribbean, South Asian/Asian/Indo-Asian, mixed, and other. In one study, the Black ethnic group was disaggregated into three different categories, but the authors did not adopt this approach for the Asian ethnic group [79]. Categorising minoritised ethnic group people into overarching categories can be useful for identifying broad patterns as some may have shared experiences of racism, discrimination, and/or social exclusion [35]. However, the use of broad ethnic categories may obscure the extent of inequalities. For example, as reported above, South Asian people may be at particular risk of MLTCs [27, 55, 80] but studies have shown that Pakistani and Bangladeshi women report higher levels of limiting long-term illness than Indian women [9]. Yet, they are often grouped together as people of South Asian ethnicity. This issue also applies to the white other ethnic group [43] who are a diverse population with particular groups, e.g. Gypsy, Roma, and Traveller community [9, 117].

#### Missing ethnicity data

The availability of complete ethnicity data is crucial for studies that seek to assess ethnic inequalities not only in relation to MLTCs, but also in relation to healthcare utilisation and care [118]. Information about the different ways in which authors handled missing ethnicity data was available in all but one of the seven studies that contributed to the evidence of ethnic inequalities in the prevalence of multiple conditions [79]. Amongst these studies, people with missing ethnicity data were excluded from analyses in three studies [27, 86, 87]. In one study, the authors used the patients’ postcode and census data to determine ethnicity [55] and in another, those with unknown ethnicity were also included in the analysis [80]. Of note is that the authors of only one study compared the sociodemographic characteristics of individuals of with missing and completed ethnicity data [75]. Researchers should carefully consider the most appropriate approach to dealing with missing data and check alternative approaches using sensitivity analysis because policies based on inaccurate data may result in poor targeting of resources and services [118].

#### Sources of data

All seven studies analysed data from patient records. For individuals to be included in patient records, they need to be in contact with healthcare services. It has been reported that access to primary care health services is generally equitable for people from minoritised ethnic groups [119]. However, they are less likely to access other specialist services [119, 120]. Therefore, those who do not access these services or for that matter, those who do not use healthcare services at all, are excluded from analyses that employ patient records. This differential access may lead to underestimation of inequalities. It is also important to consider ethnic inequalities in care quality as studies suggest that there may be differences in how patient symptoms are recorded, diagnosed or treated [121]. Other studies have not only found data quality problems when ethnicity data is recorded in hospital records (e.g. incomplete coding, inconsistent use of codes, systematic biases), but also that these data quality issues disproportionately affect hospital records for minoritised patients [122]. Consequently, the health conditions in some minoritised people may be underreported when patient records are used in analyses, thereby, impacting on the prevalence estimates of MLTCs and impeding our understanding of ethnic inequalities.

### Strengths and limitations

In presenting the state of the evidence, this review has highlighted the inconsistent ways in which MLTCs are defined and measured with the number of long-term conditions varying between studies. This lack of consensus in how MLTCs are captured has been raised by authors of other reviews of prevalence of MLTCs in the general population [123, 124]. They argue that the operational definition of multimorbidity may impact prevalence estimates and call for the standardisation of the definition and assessment of multimorbidity so as to better understand the phenomenon [123, 124]. The review has also highlighted the limitations of the studies in this area. First, many did not provide age-adjusted prevalence estimates of MLTCs. Second, not only were broad ethnic groupings used in several studies, but there were inconsistent definitions of ethnic groups. In addition, in many of these studies ethnic groups with small sample sizes and people with missing ethnicity data were often excluded from analyses. Relatedly, information on sensitivity analysis of missing ethnicity data was generally lacking. Finally, in 18% of the studies, ethnicity was assigned by practitioners, clinicians, or researchers via a combination of name recognition software and genealogy. These modes of assigning ethnicity are problematic primarily because it may be incongruent with the ways in which individuals choose to identify themselves [125]. We, therefore, have an incomplete picture of ethnic inequalities across ethnic group populations in the UK. Nonetheless, the review provides evidence of the existence of ethnic inequalities in the prevalence of multimorbidity and cardiovascular multimorbidity [27, 79, 80] prior to the COVID-19 pandemic. Studies are needed to ascertain the extent to which these inequalities have been exacerbated during the COVID19 pandemic and beyond.

A limitation of this review is that a single reviewer initially screened the titles and abstracts, and excluded irrelevant studies given the large number of studies retrieved. It is possible that some studies may have been inadvertently excluded. However, we manually searched the reference lists of key studies and relevant systematic reviews, thereby, reducing the likelihood of missing relevant studies. A strength of the review is that we conformed to the PRISMA guidelines to help transparently report the review process [23, 126] (Supplementary file 6). We also conducted the electronic search across a range of databases to identify published and unpublished studies. Not only did we assess the methodological quality, but we also assessed the relevance of the studies for understanding ethnic inequalities in the prevalence of MLTCs. Relatedly, when synthesising the results of studies that contributed to the evidence of ethnic inequalities in the prevalence of MLTCs, we only included studies that adjusted for at least age because the risk of acquiring multiple health conditions increases with age [127]. In doing so, we reduced the likelihood of presenting results that are misleading. In this review, we included studies published up until 2020 and identified three studies where COVID19 was the index condition. Whilst we do not consider COVID19 a long-term condition to be captured by definitions of multimorbidity [1, 3], we acknowledge that the pandemic has exacerbated ethnic inequalities in healthcare [128]. Our decision to focus mainly on studies published before 2020 ensures that the review sets a benchmark and provides an overview of studies that examined ethnic inequalities in the prevalence of MLTCs prior to the pandemic.

## Conclusions

In this review, we have presented the state of the evidence on ethnic inequalities in the prevalence of MLTCs in the UK prior to the COVID19 pandemic. We have identified and described the literature in this area and in doing so, illuminated the scope of work required to enhance future analyses of ethnic inequalities in people with MLTCs. With the exception of two studies that focused on diabetes, the studies identified point to the existence of ethnic inequalities in the prevalence of MLTCs. These studies also suggest that Indian, Pakistani, Bangladeshi, Black African, Black Caribbean, and people who identify as Black other, other Asian, and mixed may be at higher risk of MLTCs. Our assessment of the conceptualisation and operationalisation of ethnicity has revealed that the majority of these studies used broad ethnic categories in their analyses. They also focused on different health conditions. Further, they drew on patient records, thereby, excluding those who are not in contact with healthcare services. Thus, the results provide a partial picture of ethnic inequalities in the prevalence of multiple conditions.

### Future recommendations

The COVID19 pandemic has not only exposed structural inequalities in our society but it has also exacerbated them, especially among minoritised ethnic group people [129]. Therefore, reviews that explicitly examine the impact of the COVID19 pandemic on health(care) and quality outcomes are required to better understand the ways in which ethnic inequalities have been exacerbated and to identify potential solutions. Given the complexity of multiple conditions, the diversity of the minoritised ethnic group populations in the UK, and the varied pathways through which they come to develop MLTCs, future studies would benefit from conceptualising and analysing the prevalence of ethnic inequalities through an intersectional lens. This work could shed light on the extent to which key explanatory pathways, including racism and discrimination, play a role in the development of MLTCs in different ethnic group populations. It is findings of analyses such as these that could help to inform strategies to reduce ethnic inequalities in the prevalence of MLTCs.

## Supplementary Information

**Additional file 1.**

## Data Availability

The data extracted from the included studies are publicly available.

## Abbreviations

ASSIA: Applied Social Sciences Index and Abstracts
BAME: Black Asian and Minority Ethnic
BME: Black and Minority Ethnic
BMI: Body Mass Index
CI: Confidence Interval
COVID-19: Coronavirus Disease 2019
CPRD: Clinical Practice Research Datalink
EMBASE: Excerpta Medica data base
MLTCs: Multiple long-term conditions
NHS: National Health Service
OR: Odds Ratios
PRISMA: Preferred Reporting Items for Systematic Reviews and Meta-Analyses
THIN: The Health Improvement Network
UK: United Kingdom
