# Supplementary material for: Ethnic inequalities in multiple long-term health conditions in the United Kingdom: a systematic review and narrative synthesis
Source: BMC Public Health. 2023 Jan 27;23:178. doi: 10.1186/s12889-022-14940-w (PMC9879746; doi:10.1186/s12889-022-14940-w)
Supplement: Supplementary file 1 — Additional file 1. [file 12889_2022_14940_MOESM1_ESM.pdf]

SUPPLEMENTARY FILE 1: SEARCH TERMS

APPLIED SOCIAL SCIENCES INDEX AND ABSTRACTS

MLTCs + Ethnicity + Country	(#1 AND #2 AND #3) NOT #4 ((MAINSUBJECT.EXACT.EXPLODE("Mixed ethnicity") OR MAINSUBJECT.EXACT.EXPLODE("Ethnicity") OR ab,ti,if("Ethnic Group?" OR "african continental ancestry group" OR Arab OR Africa? OR Afro? OR Asian OR "Asian Continental Ancestry Group" OR "Asylum seeker" OR Bangladesh? OR Black OR "BME" OR "BAME" OR Caribbean OR China OR Chinese OR Cultur? OR Divers? OR Ethnic? OR Gypsy OR India? OR Irish OR Migrant OR Minorit? OR Mixed OR "Mixed ethnic?" OR "Multiple ethnic?" OR Multi$rac? OR 'Other White' OR Pakistan? OR Roma OR "White Other" OR Refugee? OR race OR racial? OR "South Asian" OR "European Continental Ancestry Group")) AND (MAINSUBJECT.EXACT("England and Wales") OR MAINSUBJECT.EXACT("Channel Islands") OR MAINSUBJECT.EXACT("UK") OR MAINSUBJECT.EXACT("Scotland") OR MAINSUBJECT.EXACT("England") OR MAINSUBJECT.EXACT("Northern Ireland") OR MAINSUBJECT.EXACT("Wales") OR ti,ab,if("United Kingdom" OR "UK" OR England OR Wales OR Scotland OR "Northern Ireland" OR Britain OR "Great Britain")) AND (ti,ab,if("Multiple Chronic Conditions" OR Co$morbid? OR Multi$morbidity OR Multi$patholog? OR "multiple condition?" OR "Multiple health condition?" OR "Multiple health problems" OR "Multiple medical conditions" OR "Multiple medical problems" OR "Pluri$patholog?" OR Polymorbid? OR "multiple illness?" OR "Multiple Chronic Health Conditions" or "Multiple Chronic Medical Conditions" OR "multiple chronic illness?") OR MAINSUBJECT.EXACT.EXPLODE("Comorbidity"))) NOT (MAINSUBJECT.EXACT.EXPLODE("USA") OR MAINSUBJECT.EXACT.EXPLODE("North America") OR MAINSUBJECT.EXACT.EXPLODE("Canada") OR MAINSUBJECT.EXACT.EXPLODE("Australia") OR MAINSUBJECT.EXACT.EXPLODE("New Zealand") OR MAINSUBJECT.EXACT.EXPLODE("South America") OR MAINSUBJECT.EXACT.EXPLODE("Central America") OR ti,ab,if("Americas" OR "USA" OR America OR "North America" OR Canada OR Australia OR "New Zealand"))
excluded countries	MAINSUBJECT.EXACT.EXPLODE("USA") OR MAINSUBJECT.EXACT.EXPLODE("North America") OR MAINSUBJECT.EXACT.EXPLODE("Canada") OR MAINSUBJECT.EXACT.EXPLODE("Australia") OR MAINSUBJECT.EXACT.EXPLODE("New Zealand") OR MAINSUBJECT.EXACT.EXPLODE("South America") OR MAINSUBJECT.EXACT.EXPLODE("Central America") OR ti,ab,if("Americas" OR "USA" OR America OR "North America" OR Canada OR Australia OR "New Zealand")
Country	(MAINSUBJECT.EXACT("England and Wales") OR MAINSUBJECT.EXACT("Channel Islands") OR MAINSUBJECT.EXACT("UK") OR MAINSUBJECT.EXACT("Scotland") OR MAINSUBJECT.EXACT("England") OR MAINSUBJECT.EXACT("Northern Ireland") OR MAINSUBJECT.EXACT("Wales")) OR ti,ab,if("United Kingdom" OR "UK" OR England OR Wales OR Scotland OR "Northern Ireland" OR Britain OR "Great Britain")
Ethnicity	(MAINSUBJECT.EXACT.EXPLODE("Mixed ethnicity") OR MAINSUBJECT.EXACT.EXPLODE("Ethnicity")) OR ab,ti,if("Ethnic Group?" OR "african continental ancestry group" OR Arab OR Africa? OR Afro? OR Asian OR "Asian Continental Ancestry Group" OR "Asylum seeker" OR Bangladesh? OR Black OR "BME" OR "BAME" OR Caribbean OR China OR Chinese OR Cultur? OR Divers? OR Ethnic? OR Gypsy OR India? OR Irish OR Migrant OR Minorit? OR Mixed OR "Mixed ethnic?" OR "Multiple ethnic?" OR Multi$rac? OR 'Other White' OR Pakistan? OR Roma OR "White Other" OR Refugee? OR race OR racial? OR "South Asian" OR "European Continental Ancestry Group")
MLTCs	ti,ab,if("Multiple Chronic Conditions" OR Co$morbid? OR Multi$morbidity OR Multi$patholog? OR "multiple condition?" OR "Multiple health condition?" OR "Multiple health problems" OR "Multiple medical conditions" OR "Multiple medical problems" OR "Pluri$patholog?" OR Polymorbid? OR "multiple illness?" OR "Multiple Chronic Health Conditions" or "Multiple Chronic Medical Conditions" OR "multiple chronic illness?") OR MAINSUBJECT.EXACT.EXPLODE("Comorbidity")

COCHRANE LIBRARY

MLTCs + Ethnicity + Country	(#1 AND #2 AND #3) NOT #4
excluded countries	"Americas" OR “USA” OR America OR “North America” OR Canada OR Australia OR “New Zealand”
Country	"United Kingdom" OR “UK” OR England OR Wales OR Scotland OR “Northern Ireland” OR Britain OR “Great Britain” OR "channel Islands"
Ethnicity	"Ethnic Group*" OR "african continental ancestry group" OR Arab OR Africa* OR Afro* OR Asian OR "Asian Continental Ancestry Group" OR “Asylum seeker” OR Bangladesh* OR Black OR “BME” OR “BAME” OR Caribbean OR China OR Chinese OR Cultur* OR Divers* OR Ethnic* OR Gypsy OR India* OR Irish OR Migrant OR Minorit* OR Mixed OR “Mixed ethnic*” OR “Multiple ethnic*” OR Multirac* OR ‘Other White’ OR Pakistan? OR Roma OR “White Other” OR Refugee* OR race OR racial* OR “South Asian” OR "European Continental Ancestry Group"
MLTCs	“Multiple Chronic Conditions” OR Comorbid* OR Multimorbidity OR Multipatholog* OR “multiple condition*” OR “Multiple health condition*” OR “Multiple health problems” OR “Multiple medical conditions” OR “Multiple medical problems” OR “Pluripatholog*” OR Polymorbid* OR “multiple illness*” OR “Multiple Chronic Health Conditions” or “Multiple Chronic Medical Conditions” OR “multiple chronic illness*”

EMBASE, MEDLINE, PsycINFO

MLTCs + Ethnicity + Country	(#1 AND #2 AND #3) NOT #4
Country	(United Kingdom) OR (UK) OR England OR Wales OR Scotland OR (Northern Ireland) OR Britain OR (Great Britain) OR (Channel Islands) OR (England adj2 Wales)
Ethnicity	(Ethnic Group*) OR (african continental ancestry group) OR Arab OR Africa* OR Afro* OR Asian OR (Asian Continental Ancestry Group) OR (Asylum seeker) OR Bangladesh* OR Black OR (BME) OR (BAME) OR Caribbean OR China OR Chinese OR Cultur* OR Divers* OR Ethnic* OR Gypsy OR India* OR Irish OR Migrant OR Minorit* OR Mixed OR (Mixed ethnic*) OR (Multiple ethnic*) OR Multirac* OR (Other White) OR Pakistan* OR Roma OR (White Other) OR Refugee* OR race OR racial* OR (South Asian) OR (European Continental Ancestry Group)
MLTCs	(Multiple Chronic Conditions) OR Comorbid* OR Multimorbidity OR Multipatholog* OR (multiple adj2 condition*) OR (Multiple health condition*) OR (Multiple health problem*) OR (Multiple medical condition*) OR (Multiple medical problems) OR Pluripatholog* OR Polymorbid* OR (multiple illness*) OR (Multiple Chronic Health Conditions) or (Multiple Chronic Medical Conditions) OR (multiple chronic illness*) OR (multiple adj2 problem) OR (multiple adj2 illness)

PUBMED

MLTCs + Ethnicity + Country	(#1 AND #2 AND #3) NOT #4 (("Multiple Chronic Conditions"[MeSH Terms] OR "Comorbidity"[MeSH Terms] OR "Multimorbidity"[MeSH Terms] OR "comorbid*"[Title/Abstract] OR "co morbid*"[Title/Abstract] OR "multimorbid*"[Title/Abstract] OR "multipatholog*"[Title/Abstract] OR "multi patholog*"[Title/Abstract] OR "multiple condition"[Title/Abstract] OR "Multiple health problems"[Title/Abstract] OR "Multiple medical conditions"[Title/Abstract] OR "Multiple medical problems"[Title/Abstract] OR "pluripatholog*"[Title/Abstract] OR "polymorbid*"[Title/Abstract] OR "multiple illness*"[Title/Abstract] OR "Multiple Chronic Health Conditions"[Title/Abstract] OR "Multiple Chronic Medical Conditions"[Title/Abstract] OR "multiple chronic illness*"[Title/Abstract]) AND ("Ethnic Groups"[MeSH Terms] OR "african continental ancestry group"[MeSH Terms] OR ("arabs"[MeSH Terms] OR "arabs"[Title/Abstract] OR "arab"[Title/Abstract]) OR

	"africa*"[Title/Abstract] OR "afro*"[Title/Abstract] OR ("Asian Continental Ancestry Group"[MeSH Terms] OR ("asian"[Title/Abstract] AND "continental"[Title/Abstract] AND "ancestry"[Title/Abstract] AND "group"[Title/Abstract]) OR "Asian Continental Ancestry Group"[Title/Abstract] OR "asian"[Title/Abstract] OR "asians"[Title/Abstract]) OR "Asian Continental Ancestry Group"[MeSH Terms] OR ("Refugees"[MeSH Terms] OR "Refugees"[Title/Abstract] OR ("asylum"[Title/Abstract] AND "seeker"[Title/Abstract]) OR "asylum seeker"[Title/Abstract]) OR ("benzoyl l arginine methyl ester"[Supplementary Concept] OR "BME"[Title/Abstract] OR "BAME"[Title/Abstract]) OR "bangladesh*"[Title/Abstract] OR ("african continental ancestry group"[MeSH Terms] OR ("african"[Title/Abstract] AND "continental"[Title/Abstract] AND "ancestry"[Title/Abstract] AND "group"[Title/Abstract]) OR "african continental ancestry group"[Title/Abstract] OR "black"[Title/Abstract] OR "african americans"[MeSH Terms] OR ("african"[Title/Abstract] AND "americans"[Title/Abstract]) OR "african americans"[Title/Abstract] OR "blacks"[Title/Abstract] OR "blackness"[Title/Abstract]) OR "BME"[Title/Abstract] OR "BAME"[Title/Abstract] OR ("caribbean s"[Title/Abstract] OR "caribbeans"[Title/Abstract] OR "west indies"[MeSH Terms] OR ("west"[Title/Abstract] AND "indies"[Title/Abstract]) OR "west indies"[Title/Abstract] OR "caribbean"[Title/Abstract] OR "caribbean region"[MeSH Terms] OR ("caribbean"[Title/Abstract] AND "region"[Title/Abstract]) OR "caribbean region"[Title/Abstract]) OR ("china"[MeSH Terms] OR "china"[Title/Abstract] OR "china s"[Title/Abstract] OR "chinas"[Title/Abstract]) OR ("Asian Continental Ancestry Group"[MeSH Terms] OR ("asian"[Title/Abstract] AND "continental"[Title/Abstract] AND "ancestry"[Title/Abstract] AND "group"[Title/Abstract]) OR "Asian Continental Ancestry Group"[Title/Abstract] OR "chinese"[Title/Abstract] OR "chineses"[Title/Abstract]) OR "cultur*"[Title/Abstract] OR "divers*"[Title/Abstract] OR ("Ethnic Groups"[MeSH Terms] OR ("ethnic"[Title/Abstract] AND "groups"[Title/Abstract]) OR "Ethnic Groups"[Title/Abstract] OR ("ethnic"[Title/Abstract] AND "group"[Title/Abstract]) OR "ethnic group"[Title/Abstract]) OR "ethnic*"[Title/Abstract] OR ("roma"[MeSH Terms] OR "roma"[Title/Abstract] OR "gypsies"[Title/Abstract] OR "gypsy"[Title/Abstract]) OR "india*"[Title/Abstract] OR "Irish"[Title/Abstract] OR ("migrant s"[Title/Abstract] OR "transients and migrants"[MeSH Terms] OR ("transients"[Title/Abstract] AND "migrants"[Title/Abstract]) OR "transients and migrants"[Title/Abstract] OR "migrant"[Title/Abstract] OR "migrants"[Title/Abstract]) OR "minorit*"[Title/Abstract] OR ("mixed"[Title/Abstract] OR "mixes"[Title/Abstract] OR "mixing"[Title/Abstract] OR "mixings"[Title/Abstract]) OR ("mixed"[Title/Abstract] OR "mixes"[Title/Abstract] OR "mixing"[Title/Abstract] OR "mixings"[Title/Abstract]) OR ("multiple"[Title/Abstract] OR "multiples"[Title/Abstract]) OR "multirac*"[Title/Abstract] OR "multi rac*"[Title/Abstract] OR ("other"[Title/Abstract] AND ("European Continental Ancestry Group"[MeSH Terms] OR ("european"[Title/Abstract] AND "continental"[Title/Abstract] AND "ancestry"[Title/Abstract] AND "group"[Title/Abstract]) OR "European Continental Ancestry Group"[Title/Abstract] OR "white"[Title/Abstract] OR "whites"[Title/Abstract])) OR "pakistan*"[Title/Abstract] OR ("roma"[MeSH Terms] OR "roma"[Title/Abstract]) OR (("European Continental Ancestry Group"[MeSH Terms] OR ("european"[Title/Abstract] AND "continental"[Title/Abstract] AND "ancestry"[Title/Abstract] AND "group"[Title/Abstract]) OR "European Continental Ancestry Group"[Title/Abstract] OR "white"[Title/Abstract] OR "whites"[Title/Abstract]) AND "other"[Title/Abstract]) OR "Refugees"[MeSH Terms] OR ("continental population groups"[MeSH Terms] OR ("continental"[Title/Abstract] AND "population"[Title/Abstract] AND "groups"[Title/Abstract]) OR "continental population groups"[Title/Abstract] OR "race"[Title/Abstract]) OR "racial*"[Title/Abstract] OR "South Asian"[Title/Abstract] OR "European Continental Ancestry Group"[MeSH Terms]) AND ("United Kingdom"[MeSH Terms] OR "UK"[Title/Abstract] OR "england"[MeSH Terms] OR "england"[Title/Abstract] OR "england s"[Title/Abstract] OR "englands"[Title/Abstract] OR "wales"[MeSH Terms] OR "wales"[Title/Abstract] OR "wales s"[Title/Abstract] OR "scotland"[MeSH Terms] OR "scotland"[Title/Abstract] OR "scotland s"[Title/Abstract] OR "Northern Ireland"[Title/Abstract] OR "britain"[Title/Abstract] OR "britain s"[Title/Abstract] OR "britains"[Title/Abstract] OR "Great Britain"[Title/Abstract])) NOT ("Americas"[MeSH Terms] OR "USA"[Title/Abstract] OR "America"[Title/Abstract] OR "North America"[Title/Abstract] OR "Canada"[Title/Abstract] OR "Australia"[Title/Abstract] OR "New Zealand"[Title/Abstract])
excluded countries	"Americas"[Mesh] OR "USA" [Title/Abstract] OR America [Title/Abstract] OR "North America" [Title/Abstract] OR Canada [Title/Abstract] OR Australia [Title/Abstract] OR "New Zealand" [Title/Abstract]
Country	"United Kingdom"[MeSH Terms] OR "UK"[Title/Abstract] OR "england"[MeSH Terms] OR "england"[Title/Abstract] OR "england s"[Title/Abstract] OR "englands"[Title/Abstract] OR

	"wales"[MeSH Terms] OR "wales"[Title/Abstract] OR "wales s"[Title/Abstract] OR "scotland"[MeSH Terms] OR "scotland"[Title/Abstract] OR "scotland s"[Title/Abstract] OR "Northern Ireland"[Title/Abstract] OR "britain"[Title/Abstract] OR "britain s"[Title/Abstract] OR "britains"[Title/Abstract] OR "Great Britain"[Title/Abstract]
Ethnicity	"Ethnic Groups"[MeSH Terms] OR "african continental ancestry group"[MeSH Terms] OR ("arabs"[MeSH Terms] OR "arabs"[Title/Abstract] OR "arab"[Title/Abstract]) OR "africa*"[Title/Abstract] OR "afro*"[Title/Abstract] OR ("Asian Continental Ancestry Group"[MeSH Terms] OR ("asian"[Title/Abstract] AND "continental"[Title/Abstract] AND "ancestry"[Title/Abstract] AND "group"[Title/Abstract]) OR "Asian Continental Ancestry Group"[Title/Abstract] OR "asian"[Title/Abstract] OR "asians"[Title/Abstract]) OR "Asian Continental Ancestry Group"[MeSH Terms] OR ("Refugees"[MeSH Terms] OR "Refugees"[Title/Abstract] OR ("asylum"[Title/Abstract] AND "seeker"[Title/Abstract]) OR "asylum seeker"[Title/Abstract]) OR ("benzoyl l arginine methyl ester"[Supplementary Concept] OR "BME"[Title/Abstract] OR "BAME"[Title/Abstract]) OR "bangladesh*"[Title/Abstract] OR ("african continental ancestry group"[MeSH Terms] OR ("african"[Title/Abstract] AND "continental"[Title/Abstract] AND "ancestry"[Title/Abstract] AND "group"[Title/Abstract]) OR "african continental ancestry group"[Title/Abstract] OR "black"[Title/Abstract] OR "african americans"[MeSH Terms] OR ("african"[Title/Abstract] AND "americans"[Title/Abstract]) OR "african americans"[Title/Abstract] OR "blacks"[Title/Abstract] OR "blackness"[Title/Abstract]) OR "BME"[Title/Abstract] OR "BAME"[Title/Abstract] OR ("caribbean s"[Title/Abstract] OR "caribbeans"[Title/Abstract] OR "west indies"[MeSH Terms] OR ("west"[Title/Abstract] AND "indies"[Title/Abstract]) OR "west indies"[Title/Abstract] OR "caribbean"[Title/Abstract] OR "caribbean region"[MeSH Terms] OR ("caribbean"[Title/Abstract] AND "region"[Title/Abstract]) OR "caribbean region"[Title/Abstract]) OR ("china"[MeSH Terms] OR "china"[Title/Abstract] OR "china s"[Title/Abstract] OR "chinas"[Title/Abstract]) OR ("Asian Continental Ancestry Group"[MeSH Terms] OR ("asian"[Title/Abstract] AND "continental"[Title/Abstract] AND "ancestry"[Title/Abstract] AND "group"[Title/Abstract]) OR "Asian Continental Ancestry Group"[Title/Abstract] OR "chinese"[Title/Abstract] OR "chineses"[Title/Abstract]) OR "cultur*"[Title/Abstract] OR "divers*"[Title/Abstract] OR ("Ethnic Groups"[MeSH Terms] OR ("ethnic"[Title/Abstract] AND "groups"[Title/Abstract]) OR "Ethnic Groups"[Title/Abstract] OR ("ethnic"[Title/Abstract] AND "group"[Title/Abstract]) OR "ethnic group"[Title/Abstract]) OR "ethnic*"[Title/Abstract] OR ("roma"[MeSH Terms] OR "roma"[Title/Abstract] OR "gypsies"[Title/Abstract] OR "gypsy"[Title/Abstract]) OR "india*"[Title/Abstract] OR "Irish"[Title/Abstract] OR ("migrant s"[Title/Abstract] OR "transients and migrants"[MeSH Terms] OR ("transients"[Title/Abstract] AND "migrants"[Title/Abstract]) OR "transients and migrants"[Title/Abstract] OR "migrant"[Title/Abstract] OR "migrants"[Title/Abstract]) OR "minorit*"[Title/Abstract] OR ("mixed"[Title/Abstract] OR "mixes"[Title/Abstract] OR "mixing"[Title/Abstract] OR "mixings"[Title/Abstract]) OR ("mixed"[Title/Abstract] OR "mixes"[Title/Abstract] OR "mixing"[Title/Abstract] OR "mixings"[Title/Abstract]) OR ("multiple"[Title/Abstract] OR "multiples"[Title/Abstract]) OR "multirac*"[Title/Abstract] OR "multi rac*"[Title/Abstract] OR ("other"[Title/Abstract] AND ("European Continental Ancestry Group"[MeSH Terms] OR ("european"[Title/Abstract] AND "continental"[Title/Abstract] AND "ancestry"[Title/Abstract] AND "group"[Title/Abstract]) OR "European Continental Ancestry Group"[Title/Abstract] OR "white"[Title/Abstract] OR "whites"[Title/Abstract])) OR "pakistan*"[Title/Abstract] OR ("roma"[MeSH Terms] OR "roma"[Title/Abstract]) OR (("European Continental Ancestry Group"[MeSH Terms] OR ("european"[Title/Abstract] AND "continental"[Title/Abstract] AND "ancestry"[Title/Abstract] AND "group"[Title/Abstract]) OR "European Continental Ancestry Group"[Title/Abstract] OR "white"[Title/Abstract] OR "whites"[Title/Abstract]) AND "other"[Title/Abstract]) OR "Refugees"[MeSH Terms] OR ("continental population groups"[MeSH Terms] OR ("continental"[Title/Abstract] AND "population"[Title/Abstract] AND "groups"[Title/Abstract]) OR "continental population groups"[Title/Abstract] OR "race"[Title/Abstract]) OR "racial*"[Title/Abstract] OR "South Asian"[Title/Abstract] OR "European Continental Ancestry Group"[MeSH Terms]
MLTCs	"Multiple Chronic Conditions"[MeSH Terms] OR "Comorbidity"[MeSH Terms] OR "Multimorbidity"[MeSH Terms] OR "comorbid*"[Title/Abstract] OR "co morbid*"[Title/Abstract] OR "multimorbid*"[Title/Abstract] OR "multipatholog*"[Title/Abstract] OR "multi patholog*"[Title/Abstract] OR "multiple condition"[Title/Abstract] OR "Multiple health condition"[Title/Abstract] OR "Multiple health problems"[Title/Abstract] OR "Multiple medical conditions"[Title/Abstract] OR "Multiple medical problems"[Title/Abstract] OR "pluripatholog*"[Title/Abstract] OR "polymorbid*"[Title/Abstract] OR "multiple

	illness*"[Title/Abstract] OR "Multiple Chronic Health Conditions"[Title/Abstract] OR "Multiple Chronic Medical Conditions"[Title/Abstract] OR "multiple chronic illness*"[Title/Abstract]

SCIENCE DIRECT

Multimorbidity + Ethnicity + Country	(Comorbid OR Multimorbid OR "multiple health conditions" OR polymorbid) AND (ethnic OR race) AND ("United kingdom" OR "Great Britain")

SCOPUS

MLTCs + Ethnicity + Country	(#1 AND #2 AND #3) NOT #4 ((TITLE-ABS-KEY (("multiple chronic conditions") OR comorbid* OR multimorbidity OR multipatholog* OR ("multiple condition*") OR ("multiple health condition*") OR ("multiple health problem*") OR ("multiple medical condition*") OR ("multiple medical problems") OR pluripatholog* OR polymorbid* OR ("multiple W/1 illness*") OR ("multiple chronic health conditions") OR ("multiple chronic medical conditions") OR ("multiple chronic illness*") OR ("multiple PRE/2 problem") OR ("multiple PRE/2 illness"))) AND (TITLE-ABS-KEY (("ethnic group*") OR ("african continental ancestry group") OR arab OR africa* OR afro* OR asian OR ("asian continental ancestry group") OR ("asylum seeker") OR bangladesh* OR black OR (bme) OR (bame) OR caribbean OR china OR chinese OR cultur* OR divers* OR ethnic* OR gypsy OR india* OR irish OR migrant OR minorit* OR mixed OR ("mixed ethnic*") OR ("multiple ethnic*") OR multirac* OR ("other white") OR pakistan* OR roma OR (white W/1 other) OR refugee* OR race OR racial* OR ("south Asian") OR ("european continental ancestry group"))) AND (TITLE-ABS-KEY (("united kingdom") OR (uk) OR england OR wales OR scotland OR ("northern Ireland") OR britain OR ("great Britain") OR ("channel islands") OR ("england and wales")))) AND NOT (TITLE-ABS-KEY (americas OR ("USA") OR america OR ("North America") OR canada OR australia OR ("New Zealand")))
excluded countries	TITLE-ABS-KEY (Americas OR ("USA") OR America OR ("North America") OR Canada OR Australia OR ("New Zealand"))
Country	TITLE-ABS-KEY (("united kingdom") OR (uk) OR england OR wales OR scotland OR ("northern Ireland") OR britain OR ("great Britain") OR ("channel islands") OR ("england and wales"))
Ethnicity	TITLE-ABS-KEY (("ethnic group*") OR ("african continental ancestry group") OR arab OR africa* OR afro* OR asian OR ("asian continental ancestry group") OR ("asylum seeker") OR bangladesh* OR black OR (bme) OR (bame) OR caribbean OR china OR chinese OR cultur* OR divers* OR ethnic* OR gypsy OR india* OR irish OR migrant OR minorit* OR mixed OR ("mixed ethnic*") OR ("multiple ethnic*") OR multirac* OR ("other white") OR pakistan* OR roma OR (white W/1 other) OR refugee* OR race OR racial* OR ("south Asian") OR ("european continental ancestry group"))
MLTCs	TITLE-ABS-KEY (("multiple chronic conditions") OR comorbid* OR multimorbidity OR multipatholog* OR ("multiple condition*") OR ("multiple health condition*") OR ("multiple health problem*") OR ("multiple medical condition*") OR ("multiple medical problems") OR pluripatholog* OR polymorbid* OR ("multiple W/1 illness*") OR ("multiple chronic health conditions") OR ("multiple chronic medical conditions") OR ("multiple chronic illness*") OR ("multiple PRE/2 problem") OR ("multiple PRE/2 illness"))

WEB OF SCIENCE CORE COLLECTION

MLTCs + Ethnicity + Country	(#1 AND #2 AND #3)
Country	TOPIC: (("united kingdom") OR (uk) OR England OR wales OR scotland OR ("northern Ireland") OR britain OR ("great Britain") OR ("channel islands") OR ("england and wales")
Ethnicity	TOPIC: ((Ethnic Group*) OR (african continental ancestry group) OR Arab OR Africa* OR Afro* OR Asian OR (Asian Continental ancestry Group) OR (Asylum seeker) OR Bangladesh* OR Black OR (BME) OR (BAME) OR Caribbean OR China OR Chinese OR Cultur* OR Divers* OR Ethnic* OR Gypsy OR India* OR Irish OR Migrant OR Minorit* OR Mixed OR (Mixed ethnic*) OR (Multiple ethnic*) OR Multirac* OR (Other White) OR Pakistan* OR Roma OR (White Other) OR Refugee* OR race OR racial* OR South Asian) OR (European Continental Ancestry Group))
MLTCs	TOPIC:((Multiple Chronic Conditions) OR Comorbid* OR Multimorbidity OR Multipatholog* OR (multiple condition*) OR (Multiple health condition*) OR (Multiple health problems) OR (Multiple medical conditions) OR (Multiple medical problems) OR (Pluripatholog*) OR Polymorbid* OR (multiple illness*) OR (Multiple Chronic Health Conditions) OR (Multiple Chronic Medical Conditions) OR (multiple chronic illness*))

OPENGREY

MLTCs + Ethnicity + Country	(Comorbid OR Multimorbid OR "multiple health conditions" OR polymorbid) AND (ethnic OR race) AND ("United kingdom" OR "Great Britain")

SUPPLEMENTARY FILE 2 : QUALITY APPRAISAL PROMPTS

**Study ID:**	**Comments**
Is the research design clearly specified and appropriate for the aims and objectives of the research? Yes ☐ No☐ Unclear☐	
Do the researchers provide a clear account of the process by which their findings were reproduced? Yes ☐ No☐ Unclear☐	
Do the researchers display enough data to support their interpretations and conclusions? Yes ☐ No☐ Unclear☐	
Is the method of analysis appropriate and adequately explicated ? Yes ☐ No☐ Unclear☐	
**Overall Quality of the Study**<br>High ☐<br>Low ☐<br>Unclear ☐<br>**Justification:**	

SUPPLEMENTARY FILE 3 : QUALITY APPRAISAL

**Study ID:**<br>**Mathur et al 2020**	**Reviewer: BH**<br>**Comments**
Is the research design clearly specified and appropriate for the aims and objectives of the research?<br>Yes ☒ No☐ Unclear☐	An observational cohort study utilizing the Clinical Practice Research Datalink (CPRD) was undertaken to address the following aims<br><br>(1) Quantify ethnic differences in risk factor levels and co-morbidities at the time of initial diagnosis,<br>(2) Compare consultation rates and completeness of process of care measures between ethnic groups in the 12 months preceding type 2 diabetes diagnosis,<br>(3) Determine whether the time to initiation of therapeutic and non-therapeutic management following initial diagnosis differed by ethnic group (Page 2)
Do the researchers provide a clear account of the process by which their findings were reproduced?<br>Yes ☒ No☐ Unclear☐	The study design, population and covariates are described in sufficient detail (Page 2-3)
Do the researchers display enough data to support their interpretations and conclusions?<br>Yes ☒ No☐ Unclear☐	The findings concerning MLTCs are described on page 3 and discussed by authors on
Is the method of analysis appropriate and adequately explicated ?<br>Yes ☒ No☐ Unclear☐	*Multivariable logistic regression (for deprivation quintile, presence of pre-diabetes, family history of diabetes, gestational diabetes, family history of cardiovascular disease, any macrovascular disease, any microvascular disease, smoking status, alcohol consumption, CVD risk, and use of antihypertensive or lipid lowering drugs) and adjusted for age at diagnosis, sex, and deprivation*<br>(Page 3).
**Overall Quality of the Study**<br>High ☒<br>Low ☐<br>Unclear ☐<br>**Justification:** The authors use appropriate methods to examine ethnic difference in the prevalence of comorbidities in a population with diabetes. They use patient records and broad ethnic group categories which mask inequalities. However, they conduct sensitivity analysis and compare those with missing ethnicity data and those with complete ethnicity data.	

**Study ID: Mathur et al 2020**	**Reviewer: MS**<br>**Comments**
1. Is the research design clearly specified and appropriate for the aims and objectives of the research?<br>Yes ☒ No☐ Unclear☐	Clear and appropriate. Uses CPRD. Focuses on people with diabetes and counts comorbidities.
2. Do the researchers provide a clear account of the process by which their findings were reproduced?<br>Yes ☒ No☐ Unclear☐	Yes, code lists are shared. Inclusion criteria are clear. Method for handling multiple ethnicities clear. All other vars clearly explained.

3. Do the researchers display enough data to support their interpretations and conclusions?<br>4. Yes ☒ No☐ Unclear☐	Yes, 5 categories of ethnicity used.
5. Is the method of analysis appropriate and adequately explicated ?<br>Yes ☒ No☐ Unclear☐	Yes, unadjusted and adjusted (age, sex. IMD) risk of comorbidities presented
**Overall Quality of the Study**<br>High ☒<br>Low ☐<br>Unclear ☐<br>**Justification:** This study only considered vascular comorbidities. Age-sex only adjusted risk of comorbidity is not available.	

**Study ID:**<br>**Dorrington et al 2020**	**Reviewer: BH**<br>**Comments**
• Is the research design clearly specified and appropriate for the aims and objectives of the research?<br>Yes ☒ No☐ Unclear☐	*This is a longitudinal study using prospective data collected by Lambeth DataNet (LDN), a primary care database of all 45 general practices within the London Borough of Lambeth* (Page 2)<br>They use this design to address the following objectives to<br>-*examine the distribution of multimorbidity and first fit note receipt by age, gender, socioeconomic deprivation and self-defined ethnicity.*<br><br>-*analyse first fit note receipt by multimorbid physical and mental health disorders.*<br><br>-*examine first fit note and maybe fit use by the number of multimorbid conditions, with and without depression*. (Page 2)
• Do the researchers provide a clear account of the process by which their findings were reproduced?<br>Yes ☒ No☐ Unclear☐	They give a detailed account of the data sources, the measures, the ethics process and the operationalisation of multimorbidity and fit note receipt (Page 2-3)
• Do the researchers display enough data to support their interpretations and conclusions?<br>• Yes ☒ No☐ Unclear☐	They provide details of the likelihood of multimorbidity by ethnic group for both basic and complex multimorbidity (Page 4) in their discussion, they discuss the inequalities they identified.<br>*They focus on We found that for the Black Caribbean population, the odds of having 3 + LTCs is over three times higher than for the White population* (Page 7)
• Is the method of analysis appropriate and adequately explicated ?<br>Yes ☒ No☐ Unclear☐	They use *descriptive percentages and multinomial logistic regression (mlogit in Stata) were used to describe baseline characteristics among study patient (Page 3)*
**Overall Quality of the Study**<br>High ☒<br>Low ☐<br>Unclear ☐<br>**Justification:** This study uses patient records. The authors' methods are appropriate to address the aims of the study. They adjust for age, deprivation and disaggregate the Black ethnic group categories but not the Asian ethnic group. They also provide evidence of inequalities for different	

conceptualisations of multimorbidity i.e. 2LTCs and 3+LTCs. No information on sensitivity analysis comparing those with and without missing data

**Study ID: Dorrington et al., 2020**	**Reviewer: MS**<br>**Comments**
Is the research design clearly specified and appropriate for the aims and objectives of the research?<br>Yes ☒ No☐ Unclear☐	Clearly specified and appropriate. Uses primary care database.
Do the researchers provide a clear account of the process by which their findings were reproduced?<br>Yes ☒ No☐ Unclear☐	LTCs based on QOF conditions plus chronic pain. Other variables clearly explained. Dataset is available on application (though this wouldn't be quick or easy to access)
Do the researchers display enough data to support their interpretations and conclusions?<br>Yes ☒ No☐ Unclear☐	Yes, unadjusted and adjusted (for age, gender and deprivation) results shown. Ethnicity categories reasonably fine-grained so can distinguish Black African, Black Caribbean, Black other as well as Mixed, Other, White, and Asian
Is the method of analysis appropriate and adequately explicated ?<br>Yes ☒ No☐ Unclear☐	Yes, appropriate method clearly explained.
**Overall Quality of the Study**<br>High ☒<br>Low ☐<br>Unclear ☐<br>**Justification:** The main limitation of this study for our interests is that it shows unadjusted and fully adjusted (including adjustment for IMD) risk of MLTCs by ethnic group. Age-sex only adjusted estimates aren't available.	

**Study ID:**<br>**Ashworth et al., 2019**	**Reviewer BH**<br>**Comments**
Is the research design clearly specified and appropriate for the aims and objectives of the research?<br>Yes ☒ No☐ Unclear☐	They describe their aims as follows and use a longitudinal study based on anonymised primary care data to address these aims. This approach is appropriate for their aims<br><br>*The aim was to study the characteristics of this multi-morbidity cohort. The main objectives were to define both the sociodemographic determinants and cardiovascular risk factors associated with multimorbidity acquisition and also to determine the acquisition sequence of multimorbidity and the influence of demographic factors on this sequence* (Page 2)
Do the researchers provide a clear account of the process by which their findings were reproduced?<br>Yes ☒ No☐ Unclear☐	These are provided in the methods section which describes the data sources, their conceptualisation of multimorbidity, the variables od interest and the study setting
Do the researchers display enough data to support their interpretations and conclusions?<br>Yes ☒ No☐ Unclear☐	The authors provide multimorbidity cohort characteristics by ethnic group which meets the inclusion criteria.
Is the method of analysis appropriate and adequately explicated ?<br>Yes ☒ No☐ Unclear☐	*We analysed sociodemographic (age, gender and ethnicity), social (area-level deprivation) and cardiovascular risk factor (hypertension, moderate obesity and smoking status) data for the*

	multimorbidity cohort and general population using univariable statistical methods applied at patient level (Page2 and 3).<br><br>*Sociodemographic and cardiovascular risk factor determinants of multimorbidity were analysed using multilevel logistic regression models to model practice-level variation* (Page 3)

Overall Quality of the Study

High ☒

Low ☐

Unclear ☐

Justification: The study uses patient records. The study design, methods and aims are appropriate to address the aims of the study. Concerning the relevance for our study aims, the authors adjust for age which is of key in studies of multimorbidity given that multimorbidity is patterned by age. However, they do not discuss the implications of using broad ethnic categories. They provide the estimates of those with unknown ethnicity data alongside the other ethnic groups.

**Study ID: Ashworth et al., 2019**	**Reviewer: LB**<br>**Comments**
Is the research design clearly specified and appropriate for the aims and objectives of the research?<br>Yes ☒ No☐ Unclear☐	
Do the researchers provide a clear account of the process by which their findings were reproduced?<br>Yes ☒ No☐ Unclear☐	
Do the researchers display enough data to support their interpretations and conclusions?<br>Yes ☒ No☐ Unclear☐	
Is the method of analysis appropriate and adequately explicated ?<br>Yes ☒ No☐ Unclear☐	

Overall Quality of the Study

High ☒

Low ☐

Unclear ☐

Justification: I've ticked high quality study based on these three categories (and because the answer to the questions above is 'yes' throughout) but didn't find this study particularly good – the use of broad categories of ethnicity is a limitation, but also how I found the way it treats ethnicity, deprivation, and socially-patterned risk factors in isolation a limitation.

**Study ID:**<br>**Owusu-Adjah et al., 2018**	**Reviewer: BH**<br>**Comments**
Is the research design clearly specified and appropriate for the aims and objectives of the research?<br>Yes ☒ No☐ Unclear☐	These are clearly stated before the methods were introduced<br>*Therefore, the aims of this primary care based retrospective longitudinal case–control study were to evaluate (1) comorbidities and cardiovascular risk factors at diagnosis of T2DM in different ethnic groups, and (2) the likelihood of developing long*

	term complications by BMI categories in different ethnic groups compared to non-diabetic control
Do the researchers provide a clear account of the process by which their findings were reproduced?<br>Yes ☒ No☐ Unclear☐	The authors provide a description of their sources of data, how they selected the participants, their variables of interest and their approach to analysis. Page 2-3
Do the researchers display enough data to support their interpretations and conclusions?<br>Yes ☒ No☐ Unclear☐	The authors provide clinical and demographic characteristics of the participants. They dedicate a section on the prevalence of comorbidities at diagnosis which is of key interest. Their interpretations are appropriate based on their findings
Is the method of analysis appropriate and adequately explicated ?<br>Yes ☒ No☐ Unclear☐	*Incidence rates ratios (IRRs) for non-fatal major cardiovascular events (MACE) and chronic kidney disease (CKD) in patients with T2DM compared to controls were estimated using multivariate Mantel-Cox model. Page 1*
**Overall Quality of the Study**<br>High ☒<br>Low ☐<br>Unclear ☐<br>**Justification:** The authors use data from The Health Improvement Network. They provide a thorough description of their methods and their interpretations are supported by their findings. Only three ethnic group categories used and patients were only included if they had complete ethnicity data.	

**Study ID: Owusu Adjah et al., 2018**	**Reviewer: LB**<br>**Comments**
• Is the research design clearly specified and appropriate for the aims and objectives of the research?<br>Yes ☒ No☐ Unclear☐	
• Do the researchers provide a clear account of the process by which their findings were reproduced?<br>Yes ☒ No☐ Unclear☐	
• Do the researchers display enough data to support their interpretations and conclusions?<br>• Yes ☒ No☐ Unclear☐	
• Is the method of analysis appropriate and adequately explicated ?<br>Yes ☒ No☐ Unclear☐	
**Overall Quality of the Study**<br>High ☒<br>Low ☐<br>Unclear ☐<br>**Justification:** Use of ethnicity poor, but other criteria met.	

**Study ID:**<br>**Mathur et al 2011**	**Reviewer: BH**<br>**Comments**
Is the research design clearly specified and appropriate for the aims and objectives of the research?<br>Yes ☒ No☒ Unclear☐	The aims of the study are<br>*to establish the distribution of cardiovascular multimorbidity between ethnic groups;*<br><br>*to explore how the management of key physician-modifiable risk factors varies by both ethnicity and level of morbidity* (page 263)<br><br>The authors use a cross-sectional study design which is clearly stated and appropriate
Do the researchers provide a clear account of the process by which their findings were reproduced?<br>Yes ☒ No☐ Unclear☐	A description of the sample, conditions of interest, ethnic categories, and confounding variables is provided (Page 263-264)
Do the researchers display enough data to support their interpretations and conclusions?<br>Yes ☒ No☐ Unclear☐	The authors note the limitations of using broad ethnic group categories (page 267)
Is the method of analysis appropriate and adequately explicated ?<br>Yes ☒ No☐ Unclear☐	Logistic regression analysis was used to examine the risk of being multimorbid by ethnic group, and the control of risk factors by ethnicity and burden of cardiovascular multimorbidity
**Overall Quality of the Study**<br>High ☒<br>Low ☐<br>Unclear ☐<br>**Justification:** The authors use routinely collected clinical data. They address the key quality assurance questions in relation to the research process. They provide fully adjusted estimates only and use broad ethnic group categories but discuss the limitations of doing so in the discussion. Concerning those with missing ethnicity data: *Patients whose ethnicity could not be classified from the recorded entry due to non-response or coding error were excluded from the analysis (p263)*	

**Study ID:**<br>**Roderick et al., 2009**	**Comments**
Is the research design clearly specified and appropriate for the aims and objectives of the research?<br>Yes ☒ No☐ Unclear☐	*Prospective cohort analysis of Renal Association UK Renal* (Page 3775).
Do the researchers provide a clear account of the process by which their findings were reproduced?<br>Yes ☒ No☐ Unclear☐	The methods are detailed enough for others to replicate. They give a detailed account of the four methods they used to ascertain ethnicity (Page 3775)
Do the researchers display enough data to support their interpretations and conclusions?<br>Yes ☒ No☐ Unclear☐	Odds ratios are provided in Table 3. The inequalities in vascular disease is highlighted in the abstract but the focus of the study is on survival of patients with renal disease
Is the method of analysis appropriate and adequately explicated ?<br>Yes ☒ No☐ Unclear☐	*Logistic regression was used to assess the effect of ethnicity on dichotomous baseline variables such as*

	late referral, adjusting for age at start of RRT and gender (Page 3775)
**Overall Quality of the Study**<br>High ☒<br>Low ☐<br>Unclear ☐<br>**Justification:** The quality of the paper is high. They use data from the Renal Association UK Renal Registry. It is important to note that the focus of the study was not on multimorbidity but the authors provide the clinical characteristics of the participants before focusing on ethnic differences in survival. They use broad ethnic groups. Concerning those with missing ethnicity data, the authors determined their 2001 Census super output area using postcode of residence; if this was an area with ≥ 98% Caucasian ethnicity, we assumed these cases were Caucasian (p.3775).	

**Study ID:**<br>**Baskar et al., 2011**	**Reviewer: BH**<br>**Comments**
Is the research design clearly specified and appropriate for the aims and objectives of the research?<br>Yes ☒ No☐ Unclear☐	The study is a cross sectional study as stated in the discussion. It is appropriate for addressing the research aims which is examine the prevalence of hypertension and vascular complications amongst Afro-Caribbean (AC), Caucasian (C) and Indo-Asian (IA) ethnic subgroups (page 214)
Do the researchers provide a clear account of the process by which their findings were reproduced?<br>Yes ☒ No☐ Unclear☐	A description of the data source, participants, comorbidities, outcome measures is provided (Page 215)
Do the researchers display enough data to support their interpretations and conclusions?<br>Yes ☒ No☐ Unclear☐	The findings on diabetic complications are displayed in Table 3 and the authors discuss the ethnic inequalities identified
Is the method of analysis appropriate and adequately explicated ?<br>Yes ☒ No☐ Unclear☐	*Multivariate logistic regression analysis was used to analyse and calculate odds ratios for individual risk factors of diabetic vascular complications and hypertension* (page 215)
**Overall Quality of the Study**<br>High ☒<br>Low ☐<br>Unclear ☐<br>**Justification:** The study uses data from the diabetes register. It is not flawed methodologically, however, they use few ethnic group categories. The authors note that of the 6485 registered adult individuals (>18 years), 415 had ethnicity status data unavailable and a small minority of 23 individuals belonged to others.	

SUPPLEMENTARY FILE 4: Quality appraisal table

	Research design clearly specified and appropriate for study aims and objectives	The process by which findings were reproduced clearly stated	Enough data displayed to support interpretations and conclusions	Method of analysis appropriate and adequately explicated	**Overall Quality of the Study**
Dorrington et al., 2020 [83]	Yes	Yes	Yes	Yes	**High**
Mathur et al., 2020 [85]	Yes	Yes	Yes	Yes	**High**
Ashworth et al., 2019 [82]	Yes	Yes	Yes	Yes	**High**
Owusu Adjah et al., 2018 [87]	Yes	Yes	Yes	Yes	**High**
Mathur et al., 2011 [26]	Yes	Yes	Yes	Yes	**High**
Roderick et al., 2009 [54]	Yes	Yes	Yes	Yes	**High**
Baskar et al., 2006 [88]	Yes	Yes	Yes	Yes	**High**

SUPPLEMENTARY FILE 5 : FIGURE SHOWING THE NUMBER OF STUDIES BY THE NUMBER OF ETHNIC GROUP CATEGORIES

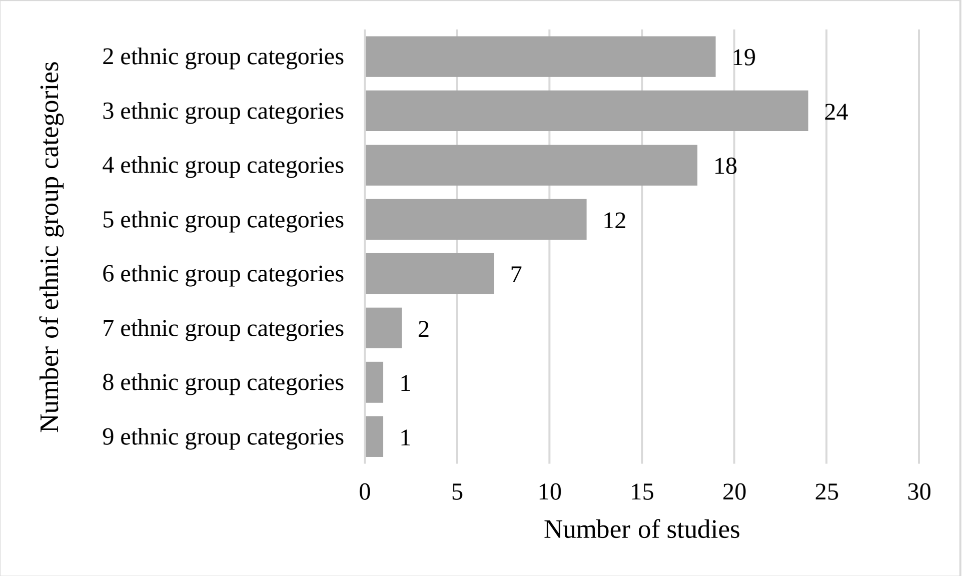

SUPPLEMENTARY FILE 6: PRISMA CHECKLIST 2020

Section and Topic	Item #	Checklist item	Location where item is reported PAGE NUMBER
**TITLE**			
Title	1	Identify the report as a systematic review.	1
**ABSTRACT**			
Abstract	2	See the PRISMA 2020 for Abstracts checklist.	1
**INTRODUCTION**			
Rationale	3	Describe the rationale for the review in the context of existing knowledge.	1, 2
Objectives	4	Provide an explicit statement of the objective(s) or question(s) the review addresses.	1, 2
**METHODS**			
Eligibility criteria	5	Specify the inclusion and exclusion criteria for the review and how studies were grouped for the syntheses.	2, 3
Information sources	6	Specify all databases, registers, websites, organisations, reference lists and other sources searched or consulted to identify studies. Specify the date when each source was last searched or consulted.	1, 2
Search strategy	7	Present the full search strategies for all databases, registers and websites, including any filters and limits used.	Supplementary file 1
Selection process	8	Specify the methods used to decide whether a study met the inclusion criteria of the review, including how many reviewers screened each record and each report retrieved, whether they worked independently, and if applicable, details of automation tools used in the process.	3
Data collection process	9	Specify the methods used to collect data from reports, including how many reviewers collected data from each report, whether they worked independently, any processes for obtaining or confirming data from study investigators, and if applicable, details of automation tools used in the process.	3
Data items	10a	List and define all outcomes for which data were sought. Specify whether all results that were compatible with each outcome domain in each study were sought (e.g. for all measures, time points, analyses), and if not, the methods used to decide which results to collect.	3
	10b	List and define all other variables for which data were sought (e.g. participant and intervention characteristics, funding sources). Describe any assumptions made about any missing or unclear information.	3
Study risk of bias assessment	11	Specify the methods used to assess risk of bias in the included studies, including details of the tool(s) used, how many reviewers assessed each study and whether they worked independently, and if applicable, details of automation tools used in the process.	3
Effect measures	12	Specify for each outcome the effect measure(s) (e.g. risk ratio, mean difference) used in the synthesis or presentation of results.	7,17
Synthesis methods	13a	Describe the processes used to decide which studies were eligible for each synthesis (e.g. tabulating the study intervention characteristics and comparing against the planned groups for each synthesis (item #5)).	3
	13b	Describe any methods required to prepare the data for presentation or synthesis, such as handling of missing summary statistics, or data	3

Section and Topic	Item #	Checklist item	Location where item is reported PAGE NUMBER
		conversions.	
	13c	Describe any methods used to tabulate or visually display results of individual studies and syntheses.	3
	13d	Describe any methods used to synthesize results and provide a rationale for the choice(s). If meta-analysis was performed, describe the model(s), method(s) to identify the presence and extent of statistical heterogeneity, and software package(s) used.	3
	13e	Describe any methods used to explore possible causes of heterogeneity among study results (e.g. subgroup analysis, meta-regression).	Narrative synthesis conducted
	13f	Describe any sensitivity analyses conducted to assess robustness of the synthesized results.	Narrative synthesis conducted
Reporting bias assessment	14	Describe any methods used to assess risk of bias due to missing results in a synthesis (arising from reporting biases).	3, Supplementary file 2 and 3
Certainty assessment	15	Describe any methods used to assess certainty (or confidence) in the body of evidence for an outcome.	3
**RESULTS**			
Study selection	16a	Describe the results of the search and selection process, from the number of records identified in the search to the number of studies included in the review, ideally using a flow diagram.	3,4
	16b	Cite studies that might appear to meet the inclusion criteria, but which were excluded, and explain why they were excluded.	3, 7
Study characteristics	17	Cite each included study and present its characteristics.	7-14
Risk of bias in studies	18	Present assessments of risk of bias for each included study.	7, 15, 16<br>Supplementary file 3 and 4
Results of individual studies	19	For all outcomes, present, for each study: (a) summary statistics for each group (where appropriate) and (b) an effect estimate and its precision (e.g. confidence/credible interval), ideally using structured tables or plots.	15 (Meta analysis not performed)
Results of syntheses	20a	For each synthesis, briefly summarise the characteristics and risk of bias among contributing studies.	15, 16
	20b	Present results of all statistical syntheses conducted. If meta-analysis was done, present for each the summary estimate and its precision (e.g. confidence/credible interval) and measures of statistical heterogeneity. If comparing groups, describe the direction of the effect.	15, 16 (Meta-Analysis not performed)

Section and Topic	Item #	Checklist item	Location where item is reported PAGE NUMBER
	20c	Present results of all investigations of possible causes of heterogeneity among study results.	Meta-Analysis not performed
	20d	Present results of all sensitivity analyses conducted to assess the robustness of the synthesized results.	Meta-Analysis not performed
Reporting biases	21	Present assessments of risk of bias due to missing results (arising from reporting biases) for each synthesis assessed.	18
Certainty of evidence	22	Present assessments of certainty (or confidence) in the body of evidence for each outcome assessed.	Supplementary file 3 and 4; 18, 19
**DISCUSSION**			
Discussion	23a	Provide a general interpretation of the results in the context of other evidence.	17,18
	23b	Discuss any limitations of the evidence included in the review.	18, 19
	23c	Discuss any limitations of the review processes used.	19, 20
	23d	Discuss implications of the results for practice, policy, and future research.	20
**OTHER INFORMATION**			
Registration and protocol	24a	Provide registration information for the review, including register name and registration number, or state that the review was not registered.	1, 2
	24b	Indicate where the review protocol can be accessed, or state that a protocol was not prepared.	1, 2
	24c	Describe and explain any amendments to information provided at registration or in the protocol.	No amendments made to protocol
Support	25	Describe sources of financial or non-financial support for the review, and the role of the funders or sponsors in the review.	20
Competing interests	26	Declare any competing interests of review authors.	21
Availability of data, code and other materials	27	Report which of the following are publicly available and where they can be found: template data collection forms; data extracted from included studies; data used for all analyses; analytic code; any other materials used in the review.	The data extracted is available in the tables. This data is also publicly available , 20
